# Transposon Insertion Site Sequencing of Providencia stuartii: Essential Genes, Fitness Factors for Catheter-Associated Urinary Tract Infection, and the Impact of Polymicrobial Infection on Fitness Requirements

**DOI:** 10.1128/mSphere.00412-20

**Published:** 2020-05-27

**Authors:** Alexandra O. Johnson, Valerie Forsyth, Sara N. Smith, Brian S. Learman, Aimee L. Brauer, Ashley N. White, Lili Zhao, Weisheng Wu, Harry L. T. Mobley, Chelsie E. Armbruster

**Affiliations:** a Department of Microbiology and Immunology, Jacobs School of Medicine and Biomedical Sciences, State University of New York at Buffalo, Buffalo, New York, USA; b Department of Microbiology and Immunology, University of Michigan Medical School, Ann Arbor, Michigan, USA; c Department of Biostatistics, University of Michigan School of Public Health, Ann Arbor, Michigan, USA; d Department of Computational Medicine & Bioinformatics, University of Michigan Medical School, Ann Arbor, Michigan, USA; University of Kentucky

**Keywords:** *Proteus mirabilis*, *Providencia stuartii*, Tn-Seq, catheter, essential genes, pathogenesis, polymicrobial, transposon, urinary tract infection

## Abstract

Providencia stuartii is a common cause of polymicrobial catheter-associated urinary tract infection (CAUTI), and yet literature describing the molecular mechanisms of its pathogenesis is limited. To identify factors important for colonization during single-species infection and during polymicrobial infection with a common cocolonizer, Proteus mirabilis, we created a saturating library of ∼50,000 transposon mutants and conducted transposon insertion site sequencing (Tn-Seq) in a murine model of CAUTI. P. stuartii strain BE2467 carries 4,398 genes, 521 of which were identified as essential for growth in laboratory medium and therefore could not be assessed for contribution to infection. Using an input/output fold change cutoff value of 20 and *P* values of <0.05, 340 genes were identified as important for establishing single-species infection only and 63 genes as uniquely important for polymicrobial infection with P. mirabilis, and 168 genes contributed to both single-species and coinfection. Seven mutants were constructed for experimental validation of the primary screen that corresponded to flagella (*fliC* mutant), twin arginine translocation (*tatC*), an ATP-dependent protease (*clpP*), d-alanine-d-alanine ligase (*ddlA*), type 3 secretion (*yscI* and *sopB*), and type VI secretion (*impJ*). Infection-specific phenotypes validated 6/7 (86%) mutants during direct cochallenge with wild-type P. stuartii and 3/5 (60%) mutants during coinfection with P. mirabilis, for a combined validation rate of 9/12 (75%). Tn-Seq therefore successfully identified genes that contribute to fitness of P. stuartii within the urinary tract, determined the impact of coinfection on fitness requirements, and added to the identification of a collection of genes that may contribute to fitness of multiple urinary tract pathogens.

**IMPORTANCE** Providencia stuartii is a common cause of polymicrobial catheter-associated urinary tract infections (CAUTIs), particularly during long-term catheterization. However, little is known regarding the pathogenesis of this organism. Using transposon insertion site sequencing (Tn-Seq), we performed a global assessment of P. stuartii fitness factors for CAUTI while simultaneously determining how coinfection with another pathogen alters fitness requirements. This approach provides four important contributions to the field: (i) the first global estimation of P. stuartii genes essential for growth in laboratory medium, (ii) identification of novel fitness factors for P. stuartii colonization of the catheterized urinary tract, (iii) identification of core fitness factors for both single-species and polymicrobial CAUTI, and (iv) assessment of conservation of fitness factors between common uropathogens. Genomewide assessment of the fitness requirements for common uropathogens during single-species and polymicrobial CAUTI thus elucidates complex interactions that contribute to disease severity and will uncover conserved targets for therapeutic intervention.

## Abstract (precis)

Providencia stuartii is a common cause of polymicrobial catheter-associated urinary tract infections (CAUTIs), particularly during long-term catheterization. However, little is known regarding the pathogenesis of this organism. Using transposon insertion site sequencing (Tn-Seq), we performed a global assessment of P. stuartii fitness factors for CAUTI while simultaneously determining how coinfection with another pathogen alters fitness requirements. This approach provides four important contributions to the field: (i) the first global estimation of P. stuartii genes essential for growth in laboratory medium, (ii) identification of novel fitness factors for P. stuartii colonization of the catheterized urinary tract, (iii) identification of core fitness factors for both single-species and polymicrobial CAUTI, and (iv) assessment of conservation of fitness factors between common uropathogens. Genomewide assessment of the fitness requirements for common uropathogens during single-species and polymicrobial CAUTI thus elucidates complex interactions that contribute to disease severity and will uncover conserved targets for therapeutic intervention.

## INTRODUCTION

Providencia stuartii is an opportunistic pathogen frequently isolated from the urine of patients with long-term indwelling urinary catheters (1–3). P. stuartii is well adapted for colonizing the catheterized urinary tract, where it persists for months (4, 5). As P. stuartii isolates are often resistant to multiple antibiotics (1, 6–8) and frequently cocolonize with other organisms (4, 5, 9–13), they contribute to the spread of antimicrobial resistance determinants to other organisms during cocolonization and to the risk of treatment failure. P. stuartii urine colonization is also a common source of bacteremia in long-term care facilities (8, 14–18). Up to 51% of P. stuartii bacteremias are polymicrobial (16, 19), suggesting that P. stuartii polymicrobial interactions promote development of bacteremia.

Despite the clinical significance of this organism, no previous studies explored P. stuartii genes essential for growth *in vitro* and little is known regarding P. stuartii virulence factors. Adherence, biofilm formation, host cell invasion, and motility are important aspects of urinary tract colonization for numerous uropathogenic bacteria, and *in vitro* and *in silico* evidence suggests that these properties may contribute to P. stuartii pathogenesis (20–26). However, their contribution to colonization and pathogenesis has not been assessed experimentally.

The polymicrobial nature of catheter-associated urinary tract infection (CAUTI) is an important consideration in investigating bacterial virulence factors. Proteus mirabilis, another common CAUTI pathogen, frequently cocolonizes the catheterized urinary tract with P. stuartii (4, 5, 9–12). In a mouse model of CAUTI, coinfection with P. stuartii and P. mirabilis increased inflammation and infection severity and altered the fitness factors required by P. mirabilis to colonize and persist within the urinary tract (23, 27). For instance, P. mirabilis did not require branched-chain amino acid biosynthesis during single-species infection, but coinfection with P. stuartii resulted in a requirement for these genes due to its high-affinity branched-chain amino acid importer (27). For common pathogens of polymicrobial infections, it is therefore imperative to define core genetic requirements for pathogenicity during both single-species infection and polymicrobial infection.

In this study, we utilized transposon insertion site sequencing (Tn-Seq) to identify P. stuartii genes essential for growth in rich medium, the full arsenal of fitness factors that contribute to single-species infection in a CAUTI model, and how fitness requirements change during polymicrobial infection. P. stuartii was found to carry a core set of 168 genes that contribute to colonization of the urinary tract during both single-species and polymicrobial infection, as well as accessory genes that contribute to only one infection type. Notably, several core genes were also identified as P. mirabilis fitness factors by Tn-Seq that were indicative of common fitness requirements for multiple urinary tract pathogens. These common fitness requirements may represent targets for treating or preventing CAUTI.

## RESULTS

### Estimation of essential genes.

**FIG 1:**
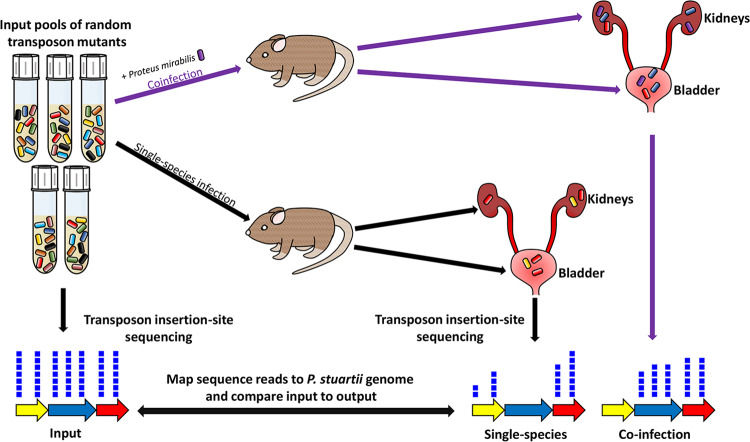
Conceptual model of single-species and polymicrobial CAUTI Tn-Seq. For each of five transposon mutant library pools, mice were infected as follows using a model of infection that includes the presence of a catheter segment within the bladder: (i) 5 to 10 CBA/J mice were transurethrally inoculated with 1 × 10^5^ CFU of the transposon library for single-species infection (black lines), and (ii) 5 to 10 CBA/J mice were inoculated with 1 × 10^5^ CFU of a 1:1 mixture of the transposon library and wild-type P. mirabilis HI4320 for coinfection (purple lines). Thus, for each input pool, the single-species infections and coinfections were conducted in parallel to utilize the same input inoculum. Input and output samples were enriched for transposon-containing sequences and subjected to next-generation Illumina sequencing of the transposon-chromosome junctions. The resulting reads were mapped to the P. stuartii BE2467 genome, and the abundances of reads at each insertion site from all output samples were compared to those determined for the input samples to determine a fold change value for each gene. The gene indicated in yellow represents a candidate P. stuartii fitness factor for single-species CAUTI that is even more important during coinfection; the gene indicated in blue represents a P. stuartii fitness factor for single-species CAUTI that is no longer important during coinfection; the gene indicated in red represents a factor that does not contribute to P. stuartii CAUTI and was therefore recovered from the infection output pools at a level of density similar to that seen with the input pools.

Based on the size of the P. stuartii BE2467 genome (4.635 Mbp, ∼4,398 genes [23]), 39,099 transposon mutants are required for >99.9% probability of full-genome coverage (28). We therefore generated five pools containing 1 × 10^4^ transposon mutants each for a total of 50,000 mutants. Insertion randomness was verified by Southern blotting (see Fig. S1 in the supplemental material). A total of 5 to 10 CBA/J mice were transurethrally inoculated with 1 × 10^5^ CFU of one of five transposon library pools for single-species infection or with 1 × 10^5^ CFU of a 1:1 mixture of the transposon library and wild-type (WT) P. mirabilis HI4320 for coinfection (Fig. 1). Thus, for each input pool, single-species infections and coinfections were conducted in parallel to utilize the same input inoculum.

10.1128/mSphere.00412-20.1 FIG S1 Verification of random transposon insertions by Southern blotting. Genomic DNA was extracted from 15 arbitrarily selected P. stuartii transposon mutants and digested with EcoRV. Samples were electrophoresed on a 1% agarose gel, which was then blotted onto a nylon membrane. Southern blotting was performed using a digoxigenin (DIG) probe against the hygromycin resistance cassette contained within the transposon. The 1,650-bp bands are part of a ladder. “+” indicates the positive-control lane derived from the pAOJ12 vector. “−” indicates the negative-control lane derived from wild-type P. stuartii BE2467 genomic DNA. Download FIG S1, TIF file, 1.5 MB. Copyright © 2020 Johnson et al. 2020 Johnson et al. This content is distributed under the terms of the Creative Commons Attribution 4.0 International license.

**FIG 2:**
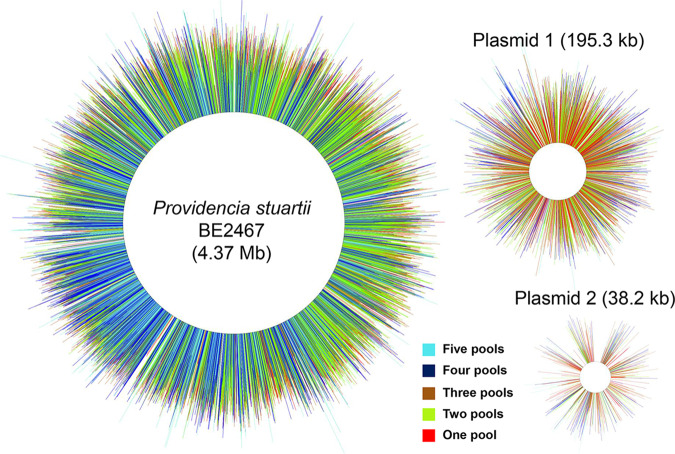
Saturation of the Providencia stuartii BE2467 genome and plasmids with transposon insertions. Chromosomal and plasmid maps indicate the location of all transposon insertions contained in all five of the input pools (not to scale). Each line represents a single insertion site from the input samples, and the length of the line represents the log_10_ number of reads recovered from each insertion site. The color of the line indicates the number of pools in which each insertion site was identified as follows: light blue, all five pools; dark blue, four pools; brown, three pools; light green, two pools; red, one pool. Regions without insertions represent estimated essential genes and intergenic regions.

Genome saturation was achieved for the P. stuartii BE2467 chromosome, plasmid PS1, and plasmid PS2, with most insertion sites represented in at least two input pools (Fig. 2). A Bayesian mixture model identified candidate genes essential for growth under laboratory conditions based on absence or underrepresentation of transposon insertions in these genes within the input pools (see Materials and Methods). The model identified 521 genes (12% of the genome) as potentially essential for growth in LB medium (see Table S1 in the supplemental material), representing a number comparable to the 436 essential genes (11.6% of the genome) of P. mirabilis HI4320 (27). Twelve of the genes estimated to be essential were carried on pPS1, including 8 hypothetical genes, an antitoxin gene (*higA*), a gene encoding a DNA-binding protein (*HU*), an arsenate reductase gene, and an arsenical resistance operon repressor; 8 were carried on pPS2, all of which are hypothetical. The potential essentiality of these genes should be interpreted with caution, as it is possible that the mariner transposon was unable to efficiently target these locations or that their loss impacts plasmid stability.

10.1128/mSphere.00412-20.5 TABLE S1 Complete Tn-Seq dataset. (First tab) Full dataset. (Second tab) Genes estimated to be essential for growth of P. stuartii in rich medium. (Third tab) P. stuartii fitness factors for colonization of the bladder and kidneys during single-species infection. (Fourth tab) P. stuartii kidney-specific fitness factors during single-species infection. (Fifth tab) P. stuartii bladder-specific fitness factors during single-species infection. (Sixth tab) P. stuartii bladder-specific fitness factors during coinfection with P. mirabilis. (Seventh tab) P. stuartii fitness factors for colonization of the bladder and kidneys during coinfection with P. mirabilis. (Eighth tab) P. stuartii kidney-specific fitness factors during coinfection with P. mirabilis. (Ninth tab) P. stuartii core fitness factors for colonization during both single-species infection and polymicrobial infection. Download Table S1, XLSX file, 1.1 MB. Copyright © 2020 Johnson et al. 2020 Johnson et al. This content is distributed under the terms of the Creative Commons Attribution 4.0 International license.

**FIG 3:**
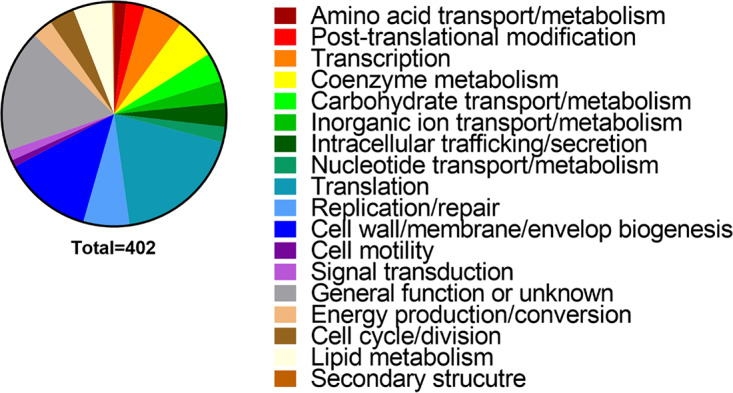
Functional categories of P. stuartii genes estimated to be essential for growth in LB medium. A total of 402 of the 521 genes estimated to be essential for growth of P. stuartii strain BE2467 had a Cluster of Orthologous Groups (COG) category. The size of each wedge of the pie chart corresponds to the percentage of genes belonging to each COG.

Among the 521 genes identified as essential, 402 could be grouped based on classification of gene category within the Clusters of Orthologous Groups (COG) database (Fig. 3), including genes associated with rRNAs, ribosomal proteins, tRNA synthetases and reductases, ATP synthase, the Sap peptide transport system, the Lol lipoprotein transport system, the Lpt lipopolysaccharide transport system, the Sec protein translocase system, and factors involved in DNA replication, cell division, and cell wall and membrane biogenesis. Several genes encoding metabolic enzymes were also identified, including the majority of the pentose phosphate pathway and phosphoglycerate kinase, which is involved in glycolysis, gluconeogenesis, and glycerol degradation. Many of the same factors were identified as essential for growth of P. mirabilis in our prior Tn-Seq study (27). Several unexpected genes were also identified, such as those encoding copper resistance (*copC*), a urea channel (*ureI*), a biofilm regulator (*bssS*), and two fimbrial chaperone proteins. While these genes may contribute to growth of P. stuartii in LB broth, another possibility is that the transposon could not efficiently target these locations for other reasons, such as a low number of TA sites or restricted access by secondary structure.

### Primary screen of P. stuartii transposon mutant libraries.

Infection studies were conducted to screen for P. stuartii fitness factors during single-species and polymicrobial CAUTI as described in the Fig. 1 legend. In all cases, a 4-mm-long segment of sterile silicone catheter tubing was inserted into the bladder during inoculation and was retained for the duration of the study (23, 27). Catheter segments were not removed prior to homogenization of bladder samples, so the CFU numbers recovered from bladder samples represent colonization of the catheter and bladder.

**FIG 4:**
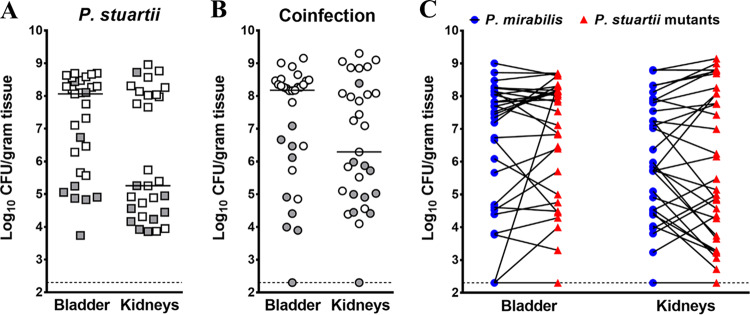
Colonization by P. stuartii transposon mutants during single-species and polymicrobial CAUTI. For each transposon mutant library pool, 5 to 10 female CBA/J mice were transurethrally inoculated with 1 × 10^5^ CFU of the transposon library for single-species infection (A), and 5 to 10 CBA/J mice were inoculated with 1 × 10^5^ CFU of a 1:1 mixture of the transposon library and wild-type P. mirabilis HI4320 for coinfection (B and C). In all cases, a 4-mm-long segment of catheter tubing was retained in the bladder for the duration of the study. Mice were sacrificed 4 days postinoculation, the bladder and kidneys were homogenized, and an aliquot was plated onto LB agar to determine bacterial burden. The remaining homogenate was fully plated for isolation of bacterial genomic DNA and sequencing. (A and B) Each symbol represents total CFU/gram of tissue from an individual mouse during single-species infection (A) or coinfection (B), and bars indicate the median. Gray-filled symbols indicate mice that were excluded from the study due to low colonization or poor quality of transposon insertion junctions during preparation for sequencing. (C) The majority of coinfected mice were highly colonized by both bacterial species; blue circles represent P. mirabilis CFU/gram of tissue, and red triangles represent P. stuartii CFU/gram of tissue, with values from a single mouse connected by a black line. Dashed lines indicate limit of detection.

Infection with P. stuartii transposon mutant pools resulted in comparable bladder and kidney bacterial burdens at 4 days postinoculation (dpi) for single-species infection and coinfection (Fig. 4A and B). The majority of coinfected mice were highly colonized by both P. stuartii and P. mirabilis, as expected (Fig. 4C). Four mice per infection type per input pool were chosen for sequencing based on colonization levels of P. stuartii and high-quality DNA during sample preparation. A fitness index was calculated for each gene as previously described (27, 29).

### P. stuartii fitness factors during single-species infection.

A total of 508 genes (11% of the genome) were identified by Tn-Seq as candidate fitness factors for single-species infection, with fold changes in insertion coverage for input/outputs ranging from 20 to 1,841 (Table S1). Among the genes, 85 encoded fitness factors for colonization of both bladder and kidneys, 414 encoded kidney-specific fitness factors, and 9 were bladder specific. Thus, P. stuartii appears to undergo an initial selection process in the catheterized bladder before ascending to the kidneys.

**FIG 5:**
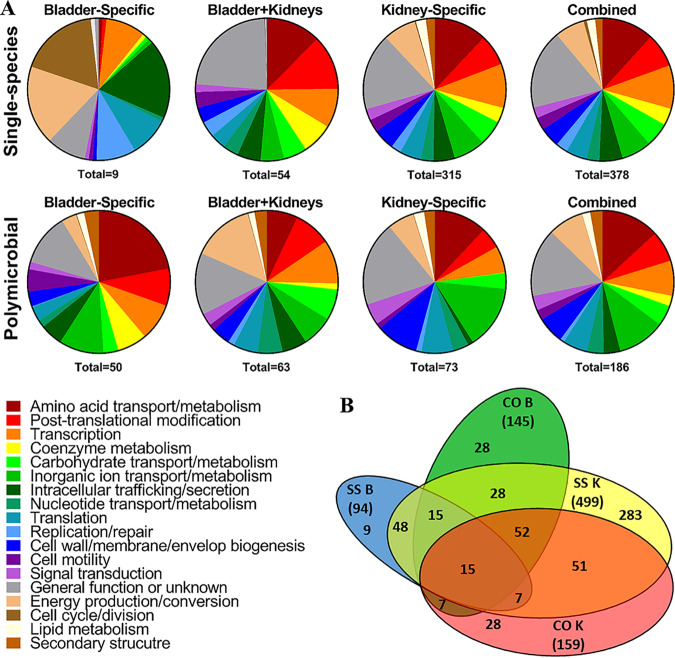
Functional categories of P. stuartii fitness factors for single-species and polymicrobial CAUTI. (A) Candidate P. stuartii fitness factors are clustered to provide an overview of the COG categories represented in each infection type and organ. The size of each pie graph wedge corresponds to the percentage of genes belonging to each COG. (B) Venn diagram showing the number of genes identified as fitness factors for each infection type and organ, including those that are not present in COG.

Among the 508 fitness factors for single-species infection, 378 were present in COG (Fig. 5A). Notably, initial Tn-Seq results confirmed our prior investigations of P. stuartii urease. Six of the eight genes in the P. stuartii plasmid-carried urease operon were underrepresented in bladder and kidney outputs compared to inputs, but the fold change values ranged from 2 to 10 and did not meet our stringent cutoff of 20. The lack of a significant fitness defect for the urease operon may have been due to the ability of other urease-positive mutants in the pool to compensate for the loss of urease activity. However, our prior study of P. stuartii CAUTI indicated that loss of the entire urease operon resulted only in a nonsignificant trend toward reduced colonization of the bladder and kidneys (23). Thus, P. stuartii urease is not critical for CAUTI in this model.

### P. stuartii fitness factors during polymicrobial infection.

Tn-Seq analyses of fitness factors from coinfection of P. stuartii BE2467 with P. mirabilis HI4320 revealed 231 candidate fitness factors (5% of the genome), with fold changes in insertion coverage for input/output ranging from 20 to 324 (Table S1). A total of 74 were important for colonization of both the catheterized bladder and the kidneys, 86 were kidney-specific fitness factors, and 71 were bladder specific (Fig. 5A). Preliminary assessment revealed concordance with our prior investigation of branched-chain amino acid import in P. stuartii. Leucine-specific binding protein LivK was previously found to contribute to bladder colonization during coinfection with P. mirabilis (27) and was identified as a fitness factor for bladder colonization during coinfection in the current study (25-fold change, *P < *0.05).

### Comparison of P. stuartii fitness factors for single-species versus polymicrobial CAUTI.

In total, 571 genes were identified by Tn-Seq as being fitness factors for P. stuartii CAUTI; 340 were specific for single-species infection (11 carried on pPS1 and 7 on pPS2), 63 were specific for polymicrobial infection (1 carried on pPS1 and 1 on pPS2), and 168 contributed to both infection types (1 carried on pPS2). A diagram of overlap of the fitness factors for each infection type and organ is presented in Fig. 5B.

Overall, there appear to be more similarities than differences in COG categories of fitness factors required by P. stuartii for single-species infection versus polymicrobial infection. One notable exception pertains to adhesins; 8 of the fitness factors specific to single-species infection encode putative fimbrial proteins and 3 of the fitness factors for both infection types were fimbrial proteins, but none were uniquely identified for coinfection. Thus, coinfection with P. mirabilis may alleviate the requirement for certain adhesins. This is in contrast to P. mirabilis, in which distinct fimbrial types were identified as fitness factors for coinfection compared to single-species infection (27). On the basis of these observations, P. mirabilis may require an array of adhesins to bind various sites within the urinary tract and form extracellular bacterial clusters and biofilms, while P. stuartii may predominantly take advantage of structures established by P. mirabilis during coinfection.

**FIG 6:**
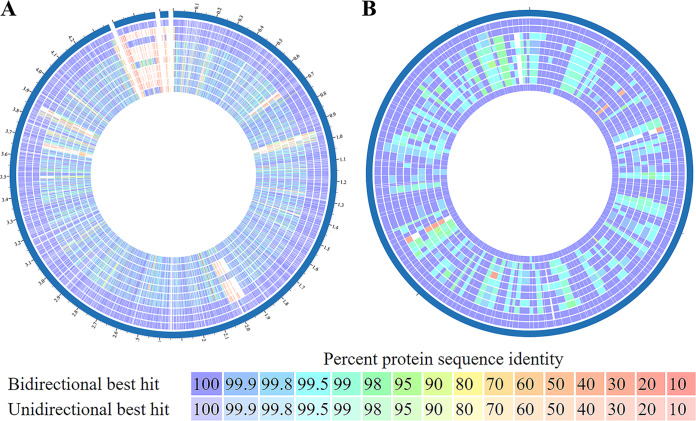
Proteome conservation between P. stuartii isolates. (A) The Pathosystems Resource Integration Center (PATRIC) Proteome Comparison tool was used to compare P. stuartii BE2467 (outer ring) with 9 other P. stuartii protein sequences, one from each node of the phylogenetic tree in Fig. S2. The three gaps in the blue location indicator separate the chromosome sequence from the two plasmid sequences. (B) A total of 166 candidate fitness factors for P. stuartii colonization of the bladder and/or kidneys during both single-species and polymicrobial CAUTI were compared across the same 9 P. stuartii isolates as follows (from outside to inside): P. stuartii BE2467, NCTC12257, 961029, ATCC 25827, FDAARGOS_87, MRSN 2154, AR_0026, INSRA21868, YD789-2, FDAARGOS_291. The color of each block indicates the protein sequence percent identity compared to P. stuartii BE2467.

The identification of 168 genes as candidate fitness factors for P. stuartii during both single-species infection and polymicrobial infection with P. mirabilis indicates that these genes likely encode critical functions for colonization of the catheterized urinary tract. For simplicity, we refer to this set of genes as “core” fitness factors to denote their importance during both single-species infection and coinfection in at least one organ (Table S1). To determine if core fitness factors are prevalent and conserved in other P. stuartii isolates, we conducted a genome comparison of P. stuartii BE2467 and 15 other P. stuartii complete genome sequences (Fig. S2). Even though P. stuartii strain BE2467 was isolated in the 1980s, there is a remarkable degree of genome conservation between this and other strains, including an isolate from 2016 (Fig. S2), and the majority of proteins encoded by P. stuartii BE2467 share ≥90% sequence identity with proteins encoded by other P. stuartii complete genome sequences (Fig. 6A). Furthermore, 164/168 core fitness factors are present with ≥95% protein sequence identity in all other P. stuartii complete genome sequences (Fig. 6B). Core fitness genes are therefore highly conserved in other P. stuartii isolates, including recent isolates from geographically distinct locations.

10.1128/mSphere.00412-20.2 FIG S2 Relatedness of P. stuartii isolates with complete genome sequences. The Pathosystems Resource Integration Center (PATRIC) Phylogenic Tree tool was used to assess the relatedness of 16 P. stuartii isolates for which a complete genome sequence was available. The tree was generated based on 100 single-copy genes, with 0 allowed deletions and 0 allowed duplications, using RAxML version 8.2.11. Download FIG S2, TIF file, 1.9 MB. Copyright © 2020 Johnson et al. 2020 Johnson et al. This content is distributed under the terms of the Creative Commons Attribution 4.0 International license.

A total of 148 of the 168 core fitness factors were also present in the genome of P. mirabilis HI4320, and 20 of these were similarly identified as P. mirabilis core fitness factors (27). These factors include a ferredoxin, an iron-sulfur cluster assembly protein (SufB), an alkyl hydroperoxide reductase, the phosphate transport system, a repressor of the glycerol-3-phosphate regulon, putrescine import, serine import, molybdenum transport, a transporter required for assembly of bd-type respiratory oxidases, and the twin arginine translocation system protein TatC. Identification of these genes as fitness factors in two uropathogens suggests that they may encode key factors required by multiple bacterial species to successfully colonize and persist within the catheterized urinary tract.

Of 168 core fitness factors identified in P. stuartii, 15 were important for colonization in both organs (bladder and kidneys) of all mice, regardless of infection type. This list includes pyruvate kinase (encoded by *pykF*), anaerobic glycerol-3-phosphate dehydrogenase (*glpB*), carbamoyl-phosphate synthase (*carA*), glycoprotein-polysaccharide metabolism (*ybaY*), the phosphotransferase system (*ptsH*), thioredoxin (*trxA*), d-alanine-d-alanine ligase (*ddlA*), ATP-dependent proteases (*clpP*), and molybdopterin synthase (*moaD*). Among the members of this list are also three core fitness factors for P. mirabilis: butanediol dehydrogenase (encoded by *butA*), d-aminoacyl-tRNA deacylase (*dtd*), and twin arginine translocation (*tatC*) (27). These 15 factors therefore pertain to critical functions required for colonization of the catheterized urinary tract, regardless of changes in the bladder environment during polymicrobial infection.

### Validation of P. stuartii candidate fitness factors for CAUTI.

**FIG 7:**
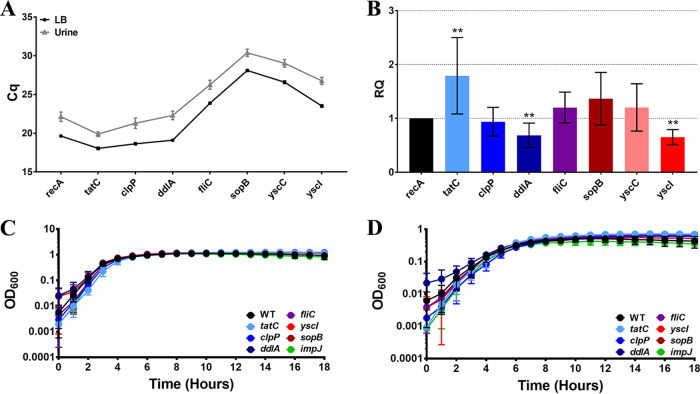
Genes of interest are expressed *in vitro* and do not significantly contribute to growth in LB broth or minimal medium. (A and B) Expression of 8 genes encoding candidate fitness factors of interest (*tatC*, *clpP*, *ddlA*, *fliC*, *sopB*, *yscC*, and *yscI*) and one housekeeping gene (*recA*) was assessed by quantitative reverse transcriptase PCR (qRT-PCR) for P. stuartii BE2467 cultured in LB broth or human urine for 2 h. (A) Raw quantification cycle (*C_q_*) values for each gene of interest. (B) The Pfaffl method was utilized to generate a Relative Quantification (RQ) value for each gene (see Materials and Methods). Error bars represent means and standard deviations of results from three independent experiments performed with two replicates each. Statistical significance was determined relative to *recA* by Wilcoxon signed-rank test (**, *P < *0.01). (C and D) Mutants were constructed in seven genes of interest, and growth was assessed in LB broth (C) and minimal medium (D) for comparison to wild-type P. stuartii BE2467. Each panel displays a representative growth curve on a log scale; error bars represent the means and standard deviations of results from four replicates.

To validate the primary screen, genes of interest were selected from following categories: (i) fitness factors for single-species infections (flagella [encoded by *fliC*]), (ii) core fitness factors for both single-species infections and polymicrobial infections (twin arginine translocation [*tatC*], an ATP-dependent protease [*clpP*], d-alanine–d-alanine ligase [*ddlA*], and type 3 secretion [T3S] [*yscI*, *yscC*, and *sopB*]), and (iii) fitness factors for polymicrobial infection (type VI secretion [*impJ*]). Expression of each gene was first assessed after a 2-h incubation in LB broth or filter-sterilized human urine, and *recA* was utilized as a housekeeping gene for normalization (Fig. 7). All genes were expressed under both sets of conditions (Fig. S3; see also Fig. 7A). Relative to *recA*, expression of *tatC* was significantly increased in urine compared to LB medium, while *ddlA* and *yscI* expression levels were significantly decreased (Fig. 7B). Mutants were generated in seven genes of interest for further validation experiments. Importantly, no mutants exhibited significant growth defects in LB medium (Fig. 7C) or minimal medium (Fig. 7D). The following subsections discuss validation of each mutant in further detail.

10.1128/mSphere.00412-20.3 FIG S3 The number of cycles between no-RT controls and test samples for qRT-PCR. The number of cycles corresponding to the point at which each primer set surpassed the threshold of detection was determined for test samples with and without reverse transcriptase. The differences in the number of cycles between RT-negative (RT^−^) and RT-positive (RT^+^) results are displayed for each primer set and for samples prepared from culture in LB medium versus urine; error bars represent means and standard deviations of results from 3 independent experiments performed with 2 replicates each. The majority of RT^+^ samples surpassed the detection threshold at least 5 cycles prior to the RT^−^ controls, with the exception of *sopB* and *yscC.* The small difference in the number of cycles determined for these primers indicates a low expression level, which can also be seen in the *C_q_* values displayed in Fig. 7A. Download FIG S3, TIF file, 0.4 MB. Copyright © 2020 Johnson et al. 2020 Johnson et al. This content is distributed under the terms of the Creative Commons Attribution 4.0 International license.

### Contribution of flagella to P. stuartii fitness during single-species CAUTI.

Flagella are well-known virulence factors of several urinary tract pathogens, including Escherichia coli and P. mirabilis, but their importance to infection has never been assessed for P. stuartii beyond a role in swarming motility *in vitro* (22). The P. stuartii BE2467 genome encodes production and secretion of flagella in a cluster of 21 genes (BGK56_00565 to BGK56_00660). The flagellar filament (*fliC*) was identified as a candidate fitness factor for kidney colonization during single-species infection, and other genes of the cluster had fitness defects in bladder colonization that ranged from 2-fold to 16-fold and kidney defects that ranged from 5-fold to 33-fold. We therefore generated a *fliC* mutant to assess the contribution of flagella to P. stuartii pathogenesis during CAUTI.

**FIG 8:**
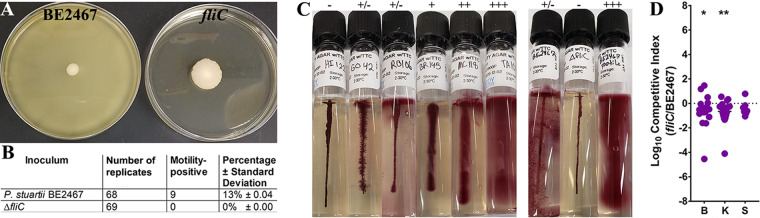
Flagella contribute to P. stuartii fitness during single-species CAUTI. (A and B) Flagellin-mediated motility was assessed for P. stuartii BE2467 and the isogenic *fliC* mutant on MOT agar. Both strains were cultured overnight in LB medium and subjected to stab inoculation into 22 to 24 MOT agar plates for each of three independent experiments. Strain BE2467 was found to be predominantly nonmotile in MOT agar, although motility was observed in 13% of replicate plates after 7 days of incubation. In contrast, motility was never detected in the *fliC* mutant. Panel A shows a representative image of a motile isolate of P. stuartii BE2467 and a typical nonmotile result from the *fliC* mutant. Panel B shows the combined number of isolates tested and the percentage that exhibited motility. (C) A panel of P. stuartii clinical isolates from the urine of catheterized nursing home residents were assessed for motility using motility test medium with triphenyltetrazolium chloride (TTC) and were compared to P. stuartii BE2467, the *fliC* mutant, and a motile revertant of BE2467. Images are representative of three independent experiments. Motility scores are indicated as follows: −, no motility; +/−, intermediate motility; +, low motility; ++, moderate motility; +++, high motility. (D) CBA/J mice (*n* = 20) were transurethrally inoculated with 1 × 10^5^ CFU of a 1:1 mixture of wild-type P. stuartii and the *fliC* mutant, and a 4-mm segment of catheter tubing was retained in the bladder for the duration of the study. After 96 h, mice were sacrificed and the catheterized bladder (B), kidneys (K), and spleen (S) were homogenized and plated onto LB agar with and without hygromycin to determine bacterial burden of wild-type P. stuartii and the mutant. A competitive index was then calculated on a per-mouse basis using the ratio of mutant to wild-type CFUs in each organ divided by the ratio of mutant to wild-type CFUs from the inoculum (see Materials and Methods). Statistical significance was assessed by Wilcoxon signed-rank test (*, *P < *0.05; **, *P < *0.01).

To assess motility in P. stuartii BE2467, bacteria were subjected to stab inoculation into soft agar plates and incubated at 37°C for 7 days. Wild-type P. stuartii BE2467 did not exhibit motility during the first ∼48 h of incubation, although motility emerged from 13% of soft agar plates by day 7 (*n* = 68), while none of the plates inoculated with the *fliC* mutant exhibited motility (*n* = 69) (Fig. 8A and B). Motility is known to be a variable phenotype in P. stuartii (2) and may be less common during long-term colonization. We therefore utilized commercial motility test medium with triphenyltetrazolium chloride (TTC) to test the motility of a panel of 17 P. stuartii isolates collected from the urine of long-term catheterized individuals during a 24-h incubation. In this medium, motile bacteria extend outward from the stab line and reduce the TTC, generating a diffuse red pigment, while nonmotile organisms grow only along the stab line, generating a concentrated line of red pigment and leaving the surrounding medium clear (Table 1; see also Fig. 8C). The clinical isolates displayed a range of motility phenotypes (Fig. 8C): 6 isolates (35%) were nonmotile; 4 (24%), including strain BE2467, exhibited intermediate motility; and 7 (41%) exhibited classic motility. Importantly, loss of *fliC* abrogated the intermediate motility of BE2467, while motile isolates recovered from the plates in Fig. 8A exhibited classic motility, suggesting that BE2467 is capable of modulating motility.

**TABLE 1:** Assessment of motility in P. stuartii clinical isolates^*a*^

Isolate ID	Motility score
BE2467	+/−
BE2467 Δ*fliC*	−
BE125	−
HI122	−
206v0MAC4	−
KU146	−
DE266	−
101v0MAC2	−
101v0MAC3	+/−
RO106	+/−
GO42	+/−
MC118	+
BR143	+
201v0MAC1	++
202v0MAC2	++
TA101	+++
NI114	+++
955	+++
BE2467 motile	+++

a Motility scores (−, no motility; +/−, intermediate motility; +, low motility; ++, moderate motility; ++, high motility) are based on three independent assessments of each isolate. ID, identifier.

The *fliA* gene sequence of wild-type P. stuartii BE2467 has a two-nucleotide insertion (TG) 468 nucleotides into the coding sequence of the gene compared to *fliA* sequence from several other P. stuartii genomes, resulting in a frameshift that may explain the intermediate motility exhibited by this strain (Fig. S4). However, a P. stuartii strain that was isolated from human urine in 2014 exhibits 100% *fliA* nucleotide identity to strain BE2467, indicating that this two-nucleotide insertion in *fliA* is not unique to BE2467. It is also notable that both motile revertants of P. stuartii BE2467 had a two-nucleotide deletion within *fliA*, shifting it back into frame (22 nucleotides downstream of the TG in isolate 1 and 3 nucleotides downstream of the TG in isolate 2, as shown in Fig. S4). Thus, P. stuartii BE2467 can spontaneously revert to classic motility *in vitro* and this may also occur for other P. stuartii isolates.

10.1128/mSphere.00412-20.4 FIG S4 Comparison of *fliA* sequences. The nucleotide sequence of the P. stuartii *fliA* gene starting at 464 nucleotides from the start of the coding sequence is presented. The *fliA* sequence in several P. stuartii isolates is 687 nucleotides, but strain BE2467 contains an insertion of 2 bp (TG) at position 468 compared to the *fliA* sequence in six other available P. stuartii genome sequences (strains MRSN, NCTC11800, 961029, AR_0026, FAARGOS_294, and ATCC 33672). This insertion causes a frameshift in the 3′ region of *fliA*, and its presence likely explains why the strain is not readily motile on MOT agar. The same insertion is also present in a urine isolate from 2014 (FAARGOS_291), indicating that this is not unique to strain BE2467. Notably, the *fliA* region from each of two motile isolates recovered after a 7-day incubation on MOT agar was also sequenced, and each exhibited a 2-bp deletion, shifting *fliA* back in frame. Download FIG S4, TIF file, 0.3 MB. Copyright © 2020 Johnson et al. 2020 Johnson et al. This content is distributed under the terms of the Creative Commons Attribution 4.0 International license.

Notably, wild-type P. stuartii BE2467 still expresses *fliC* during incubation in LB medium and urine (Fig. 7B). It has also been demonstrated in other bacterial species that flagella can have an additional role in pathogenesis that is independent of motility. For instance, in Pseudomonas aeruginosa, production of an intact flagellum is important for resistance to surfactant protein A (SP-A), which facilitates uptake of the bacterium by phagocytic cells (30). We therefore investigated the contribution of *fliC* to pathogenicity. Female CBA/J mice were transurethrally inoculated with 1 × 10^5^ CFU of a 1:1 mixture of the *fliC* mutant and wild-type P. stuartii, the catheterized bladder and kidneys were homogenized for determination of bacterial burden after 96 h, and a competitive index (CI) value was calculated (Fig. 8C). As CAUTI can progress to secondary bacteremia, the spleen was also homogenized for determination of bacterial burden as an indicator of dissemination to the bloodstream. The *fliC* mutant exhibited a significant fitness defect in the catheterized bladder and kidneys during cochallenge with the wild-type strain, confirming the results of the primary Tn-Seq screen. Thus, while *in vitro* motility is variable in P. stuartii BE2467, flagella contribute to motility in P. stuartii BE2467 CAUTI. The mutant did not exhibit a fitness defect in the spleen, indicating that flagella do not contribute to development of bacteremia or survival once within the bloodstream. Flagella similarly do not contribute to Proteus mirabilis survival in the bloodstream (29).

### Contribution of twin arginine translocation to P. stuartii fitness during single-species and polymicrobial CAUTI.

The twin arginine translocase (Tat) system is used by numerous Gram-negative bacterial species to export prefolded proteins carrying a twin arginine motif signal sequence. Tat substrates are generally periplasmic proteins and enzymes, many of which are involved in binding redox cofactors (31). The protein export system is comprised of three proteins (TatA, TatB, and TatC), of which *tatA* and *tatC* were identified as core fitness factors. Prior work in P. stuartii strain XD37 revealed that this species produces a TatA protein with an N-terminal extension that must be cleaved by rhomboid-type protease AarA to be active and that both *aarA* and *tatC* are required for production of a melanin-like pigment as well as for aerobic growth on MacConkey agar plates containing sodium deoxycholate (32). Interestingly, *aarA* (BGK56_18970) was not identified as a fitness factor in P. stuartii BE2467, likely because this strain encodes two additional rhomboid-type proteases that may also be capable of processing TatA (BGK56_13380 and BKG56_12285). We therefore generated a *tatC* mutant for validation of the importance of twin arginine translocation to P. stuartii CAUTI.

**FIG 9:**
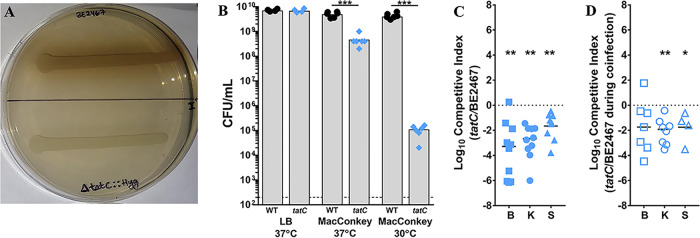
Twin arginine translocation contributes to P. stuartii fitness during single-species and polymicrobial CAUTI. P. stuartii BE2467 and the isogenic *tatC* mutant were assessed for pigment production (A), susceptibility to bile salts on MacConkey agar (B), fitness during single-species infection (C), and fitness during coinfection with P. mirabilis HI4320 (D). (A) Representative image of pigment production by wild-type P. stuartii and the *tatC* mutant on LB agar supplemented with tyrosine and iron sulfate. (B) Levels of viable P. stuartii BE2467 and the *tatC* mutant (CFU/ml) following incubation on LB or MacConkey agar at 37°C or 30°C. Gray bars represent the mean levels of CFU/ml from 3 independent experiments performed with 2 replicates each. Statistical significance was assessed by Student's *t* test (***, *P < *0.001). (C) CBA/J mice (*n* = 10) were transurethrally inoculated with 1 × 10^5^ CFU of a 1:1 mixture of wild-type P. stuartii and the *tatC* mutant, a 4-mm segment of catheter tubing was retained in the bladder for the duration of the study, and a competitive index was calculated as described above. (D) CBA/J mice (*n* = 8) were transurethrally inoculated with 1 × 10^5^ CFU of a 1:1:2 mixture of wild-type P. stuartii, the *tatC* mutant, and wild-type P. mirabilis HI4320. A competitive index was again calculated for the *tatC* mutant relative to wild-type P. stuartii BE2467 to determine fitness of the mutant during coinfection. Statistical significance was assessed by Wilcoxon signed-rank test (*, *P < *0.05; **, *P < *0.01).

The *tatC* mutant of P. stuartii BE2467 exhibited expected *in vitro* phenotypes of reduced pigment production (Fig. 9A) and a growth defect on MacConkey agar (Fig. 9B) as previously observed in P. stuartii strain XD37 (32). Upon direct competition with wild-type P. stuartii BE2467, the *tatC* mutant exhibited a significant defect in colonization of the catheterized bladder, kidneys, and spleen, indicating that Tat substrates are critical for P. stuartii fitness within the catheterized urinary tract (Fig. 9C). To determine if the Tat system also contributes to polymicrobial infection, CBA/J mice were transurethrally inoculated with 1 × 10^5^ CFU of the following mixture: 5 × 10^4^ CFU of a 1:1 mixture of the *tatC* mutant and wild-type P. stuartii and 5 × 10^4^ CFU of wild-type P. mirabilis (Fig. 9D). The *tatC* mutant exhibited significant defects during coinfection with P. mirabilis, confirming the importance of Tat substrates during both single-species infection and polymicrobial infection. The Tat system was also identified as a core fitness factor in P. mirabilis (27) and is critical for survival in the bloodstream (29). Thus, Tat substrates may be critical for the fitness of a variety of bacterial species within the urinary tract and bloodstream.

### Contribution of an ATP-dependent protease to P. stuartii fitness during single-species and polymicrobial CAUTI.

Most energy-dependent protein degradation in bacteria is mediated by five ATP-dependent proteases: FtsH (HflB), the Clp proteases (ClpAP, ClpXP, and ClpYQ/HslVU), and the Lon protease (33). P. stuartii BE2467 carries *ftsH*, *clpA*, *clpP*, *clpX*, *hslU*, and *hslV*, of which *clpP* and *hslV* were identified as core fitness factors. To validate the importance of ATP-dependent proteases to P. stuartii fitness, we generated a mutant in *clpP* to disrupt both ClpAP and ClpXP.

**FIG 10:**
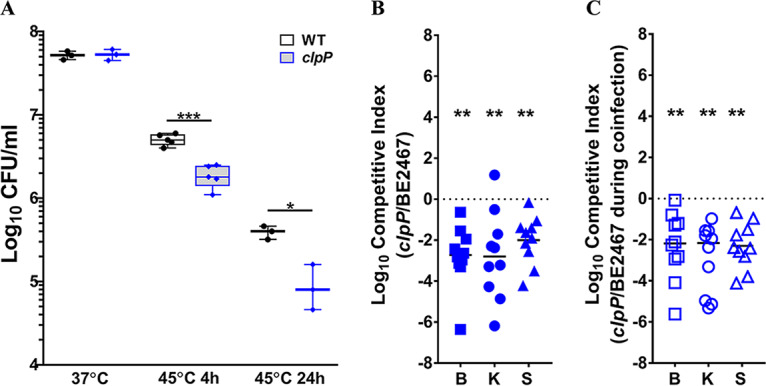
The ClpP ATP-dependent protease contributes to P. stuartii fitness during single-species and polymicrobial CAUTI. P. stuartii BE2467 and the isogenic *clpP* mutant were assessed for heat sensitivity (A), fitness during single-species infection (B), and fitness during coinfection with P. mirabilis HI4320 (C). (A) Viable CFU/ml recovered for wild-type P. stuartii BE2467 and the *clpP* mutant following incubation at 37°C versus 45°C. Box-and-whisker plots show all data points with minimum, maximum, and mean values for results from at least 3 independent experiments. (B) CBA/J mice (*n* = 10) were transurethrally inoculated with 1 × 10^5^ CFU of a 1:1 mixture of wild-type P. stuartii and the *clpP* mutant, and a competitive index was calculated as described above. (C) CBA/J mice (*n* = 10) were transurethrally inoculated with 1 × 10^5^ CFU of a 1:1:2 mixture of wild-type P. stuartii, the *clpP* mutant, and wild-type P. mirabilis HI4320, a 4-mm segment of catheter tubing was retained in the bladder for the duration of the study, and a competitive index was calculated as described above. Statistical significance was assessed by Wilcoxon signed-rank test (**, *P < *0.01).

The *clpP* mutant was assessed to determine if loss of ClpAP and/or ClpXP would impact the ability to survive heat stress (Fig. 10A). Wild-type P. stuartii and the *clpP* mutant grew comparably at 37°C, but the *clpP* mutant exhibited reduced viability during incubation at 45°C for 4 h, which became more pronounced after 24 h (Fig. 10A). Thus, loss of *clpP* results in sensitivity to heat stress.

To validate the contribution of ClpP to P. stuartii fitness during CAUTI, the *clpP* mutant was directly competed against wild-type P. stuartii during single-species infection (Fig. 10B) or during coinfection with P. mirabilis (Fig. 10C). The *clpP* mutant was significantly outcompeted in all organs during both types of infection, underscoring the central importance of ATP-dependent protein degradation to fitness during infection. This finding is notable as all five ATP-dependent protease types were found to represent either essential genes or fitness factors in our prior Tn-Seq study of P. mirabilis, suggesting that proteases are important fitness factors for numerous uropathogens (27).

### Contribution of d-alanine-d-alanine ligase to P. stuartii fitness during single-species CAUTI.

The peptidoglycan layer of the bacterial cell envelope consists of linear chains of polysaccharides cross-linked by short peptides, each unit of which is first synthesized in the cell cytoplasm and then flipped across the membrane (reviewed in reference 34). During synthesis in the cytoplasm, short peptides are capped by two d-alanine residues via the action of d-alanine-d-alanine ligase (*ddl*), and these terminal d-alanine residues are critical for peptidoglycan cross-linking by transpeptidases. The Ddl enzymes therefore serve an essential role in peptidoglycan biosynthesis, some of which provide antibiotic resistance due to altered substrate specificity (such as vancomycin-resistant Ddl enzymes VanA, VanB, and VanC) (35).

**FIG 11:**
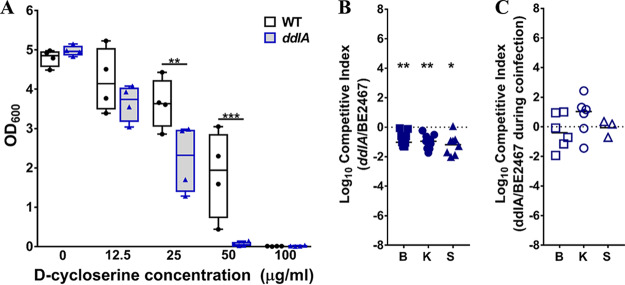
d-Alanine–d-alanine ligase contributes to P. stuartii fitness during single-species CAUTI but not during polymicrobial infection. P. stuartii BE2467 and the isogenic *ddlA* mutant were assessed for sensitivity to d-cycloserine (A), fitness during single-species infection (B), and fitness during coinfection with P. mirabilis HI4320 (C). (A) Optical density of bacterial cultures following incubation with increasing concentrations of d-cycloserine. Box-and-whisker plots show all data points with minimum, maximum, and mean values for results from 4 independent experiments. (B) CBA/J mice (*n* = 9) were transurethrally inoculated with 1 × 10^5^ CFU of a 1:1 mixture of wild-type P. stuartii and the *ddlA* mutant, and a competitive index was calculated as described above. (C) CBA/J mice (*n* = 6) were transurethrally inoculated with 1 × 10^5^ CFU of a 1:1:2 mixture of wild-type P. stuartii, the *ddlA* mutant, and wild-type P. mirabilis HI4320, a 4-mm segment of catheter tubing was retained in the bladder for the duration of the study, and a competitive index was calculated as described above. Statistical significance was assessed by Wilcoxon signed-rank test (*, *P < *0.05; **, *P < *0.01).

In our prior Tn-Seq investigation of P. mirabilis HI4320, we determined that P. mirabilis carries only one *ddl* gene (*ddlA*), which was identified as essential for growth in LB medium (27, 36). In contrast, P. stuartii BE2467 carries two *ddl* genes, one that was identified as essential for growth in LB medium (*ddlB*, BGK56_07090) and one that was identified as a candidate fitness factor (*ddlA*, BGK56_02735). We therefore generated a mutant in *ddlA*.

d-Cycloserine is a structural analog of d-alanine with antibiotic properties, as it is capable of interfering with peptidoglycan biosynthesis by inhibiting Ddl (37). We therefore hypothesized that the *ddlA* mutant would exhibit increased sensitivity to d-cycloserine compared to the wild type. Growth of the *ddlA* mutant was significantly perturbed by as little as 25 μg/ml of d-cycloserine and completely inhibited by 50 μg/ml, while growth of wild-type P. stuartii BE2467 was inhibited by 100 μg/ml (Fig. 11A). Thus, even though P. stuartii BE2467 encodes two Ddl enzymes, loss of *ddlA* has a significant phenotype *in vitro.*

To validate the contribution of DdlA to P. stuartii fitness within the catheterized urinary tract, the *ddlA* mutant was directly competed against wild-type P. stuartii during single-species infection (Fig. 11B) or during coinfection with P. mirabilis (Fig. 11C). Interestingly, loss of *ddlA* resulted in a fitness defect during single-species infection but not during coinfection. It is possible that the second *ddl* gene is capable of compensating for loss of *ddlA* during polymicrobial infection.

### Contribution of type 3 secretion to P. stuartii fitness during single-species CAUTI.

Type 3 secretion system virulence factors in numerous bacterial species are well known for their ability to inject effectors into host cells, promoting uptake of bacteria into an intracellular niche. A cluster of 20 genes in P. stuartii BE2467 are predicted to encode a type 3 secretion (T3S) system (BGK56_12745 through BGK56_12840). Three T3S genes were identified as core fitness factors (*yscF*, *yscI*, and *yscJ*/*prgK*); YscI (BGK56_12830) is predicted to be the inner rod of the T3S apparatus, YscF the needle, and YscJ/PrgK an inner membrane ring (38).

**FIG 12:**
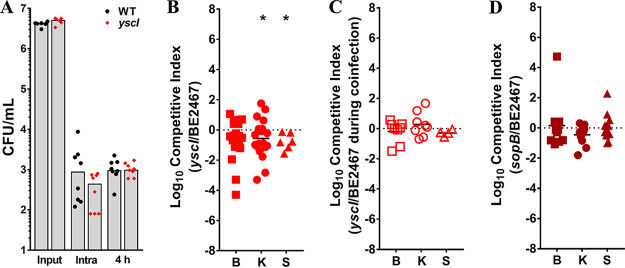
Type 3 secretion contributes to P. stuartii fitness during single-species CAUTI but not during polymicrobial infection. P. stuartii BE2467 and the isogenic *yscI* mutant were assessed for intracellular survival in RAW macrophage-like cells (A), fitness during single-species infection (B), and fitness during coinfection with P. mirabilis HI4320 (C). (A) Log_10_ CFU/ml of viable bacteria recovered from the input inoculum, intracellular bacteria 15 min after removal of extracellular bacteria via amikacin treatment, and intracellular bacteria 4 h after removal of extracellular bacteria. Gray bars indicate the means of results from 2 independent experiments performed with 4 replicates each. No significant differences were detected by a nonparametric Mann-Whitney test. (B) CBA/J mice (*n* = 20) were transurethrally inoculated with 1 × 10^5^ CFU of a 1:1 mixture of wild-type P. stuartii and the *yscI* mutant, a 4-mm segment of catheter tubing was retained in the bladder for the duration of the study, and a competitive index was calculated as described above. (C) CBA/J mice (*n* = 8) were transurethrally inoculated with 1 × 10^5^ CFU of a 1:1:2 mixture of wild-type P. stuartii, the *yscI* mutant, and wild-type P. mirabilis HI4320, and a competitive index was calculated as described above. (D) CBA/J mice (*n* = 13) were transurethrally inoculated with 1 × 10^5^ CFU of a 1:1 mixture of wild-type P. stuartii and the *sopB* mutant, and a competitive index was calculated as described above. Statistical significance was assessed by Wilcoxon signed-rank test (*, *P < *0.05; **, *P < *0.01).

The T3S components of P. stuartii BE2467 were annotated based on similarities to the T3S system of *Yersinia* species and the SPI-1 T3S system of *Salmonella* species. *Salmonella* species utilize the SPI-1 T3S system to invade nonphagocytic host cells and inhibit apoptosis, and *Yersinia* species utilize a plasmid-encoded T3S system for replication and survival inside macrophages (38). One critical factor secreted by the *Salmonella* SPI-1 T3S system is inositol phosphatase SopB, which can promote bacterial uptake by nonphagocytic cells and prolong host cell survival, allowing optimal intracellular bacterial growth (39–41). P. stuartii BE2467 encodes a *sopB* product homolog (BGK56_15180) with 48% amino acid sequence identity to that of Salmonella dublin FMB1, the species strain from which this protein was originally identified (42), and *sopB* was identified as a candidate fitness factor for kidney colonization during single-species infection. We therefore sought to determine the contribution of T3S to intracellular survival of P. stuartii in macrophages, in addition to pathogenesis *in vivo* (Fig. 12).

We first assessed the contribution of T3S to P. stuartii survival in the RAW 264.7 murine macrophage cell line. The wild-type strain and the *yscI* mutant were internalized by the RAW cells to similar degrees, and we observed no differences in intracellular survival of the mutant compared to the wild-type strain after 4 h (Fig. 12A). Thus, the T3S system of P. stuartii did not appear to contribute to uptake by phagocytic cells or survival *in vitro* under our experimental conditions.

To assess the contribution of T3S to P. stuartii fitness within the catheterized urinary tract, the *yscI* mutant was directly competed against wild-type P. stuartii during single-species infection (Fig. 12B) or during coinfection with P. mirabilis (Fig. 12C). Interestingly, loss of *yscI* resulted in a significant fitness defect only during single-species infection and not during coinfection with P. mirabilis. Thus, T3S may be dispensable during coinfection, possibly due to the increased tissue damage that occurs with P. mirabilis (23). Considering that the *yscI* mutant exhibited a significant defect in the kidney during single-species infection, we next assessed the potential contribution of the putative T3S effector *sopB* to fitness during single-species infection (Fig. 12D). However, the *sopB* mutant did not exhibit a significant fitness defect in any organ. The lack of phenotype for *sopB* during single-species infection may have been due to compensation by other T3S effectors.

### Contribution of type VI secretion to polymicrobial CAUTI.

Type VI secretion (T6S) systems are common in Gram-negative bacteria and can mediate microbe-microbe interactions as well as host-microbe interactions through injection of effector proteins. A minimum of 13 genes are required for the assembly and function of the T6S apparatus (now universally named TssA-TssM), but T6S gene clusters can contain more than 30 genes due to the presence of additional accessory factors and effectors (43, 44). The T6S gene cluster of P. stuartii BE2467 contains 18 genes (BGK56_12525 to BGK56_12610). While no single gene in the cluster met our stringent fold change cutoff, 2 genes were estimated to be essential due to a lack of transposon insertions in the input pool and the remaining 16 genes all had statistically significant (*P < *0.001) fitness defects ranging from 2.6-fold to 19.8-fold in the bladder and kidneys during both single-species and polymicrobial infection. The potential importance of T6S to P. stuartii pathogenesis is notable, as our prior CAUTI Tn-Seq study revealed that all five T6S effector operons of P. mirabilis HI4320 contained candidate fitness factors for coinfection with P. stuartii (27). We therefore sought to determine the importance of T6S to fitness of both P. stuartii and P. mirabilis during single-species and polymicrobial CAUTI.

**FIG 13:**
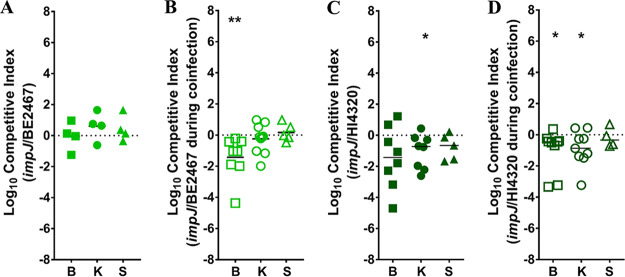
Type 6 secretion contributes to fitness of P. stuartii and P. mirabilis during polymicrobial CAUTI. The contribution of T6S to P. stuartii fitness (A and B) and P. mirabilis fitness (C and D) was assessed using isogenic *impJ* mutants of each species. (A) CBA/J mice (*n* = 4) were transurethrally inoculated with 1 × 10^5^ CFU of a 1:1 mixture of wild-type P. stuartii and its *impJ* mutant, a 4-mm segment of catheter tubing was retained in the bladder for the duration of the study, and a competitive index was calculated as described above. (B) CBA/J mice (*n* = 10) were transurethrally inoculated with 1 × 10^5^ CFU of a 1:1:2 mixture of wild-type P. stuartii, its *impJ* mutant, and wild-type P. mirabilis HI4320, and a competitive index was calculated as described above. (C) CBA/J mice (*n* = 8) were transurethrally inoculated with 1 × 10^5^ CFU of a 1:1 mixture of wild-type P. mirabilis and its isogenic *impJ* mutant, and a competitive index was calculated as described above. (D) CBA/J mice (*n* = 9) were transurethrally inoculated with 1 × 10^5^ CFU of a 1:1:2 mixture of wild-type P. mirabilis, its *impJ* mutant, and wild-type P. stuartii BE2467, and a competitive index was calculated as described above. Statistical significance was assessed by Wilcoxon signed-rank test (*, *P < *0.05; **, *P < *0.01).

ImpJ (TssK) is part of the baseplate of the T6S apparatus and essential for sheath polymerization, and disruption of *impJ* prevents T6S function (43, 45, 46). We generated an *impJ* mutant in P. stuartii BE2467 by disrupting BGK56_12575 and tested fitness during cochallenge with the parental isolate for single-species CAUTI and coinfection with P. mirabilis HI4320 (Fig. 13A and B). The P. stuartii *impJ* mutant did not exhibit an obvious fitness defect during monomicrobial infection, but it was significantly outcompeted by wild-type P. stuartii in the catheterized bladder during polymicrobial infection with wild-type P. mirabilis, suggesting that T6S contributes to P. stuartii fitness during coinfection.

To conduct the reciprocal experiment with P. mirabilis, we utilized a previously constructed mutant in P. mirabilis *impJ* (PMI0742) that was confirmed to lack T6S functionality (47). Interestingly, the P. mirabilis *impJ* mutant was outcompeted by wild-type P. mirabilis in the kidneys during single-species infection and was outcompeted in bladder and kidneys during coinfection with P. stuartii (Fig. 13C and D). Thus, T6S appears to contribute to the fitness of both bacterial species during coinfection and may contribute to P. mirabilis fitness during single-species CAUTI.

### Assessing P. stuartii candidate fitness factors by independent challenge.

**FIG 14:**
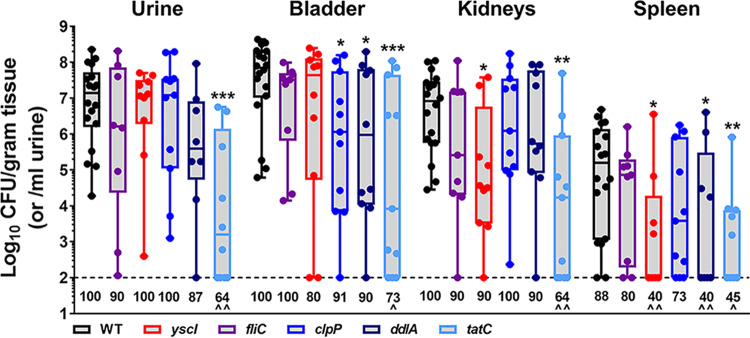
Independent challenge of candidate fitness factors. CBA/J mice were transurethrally inoculated with 1 × 10^5^ CFU of wild-type P. mirabilis or an individual mutant of interest, and a 4-mm segment of catheter tubing was retained in the bladder for the duration of the study. Mice were sacrificed 4 days postinoculation, and bacterial burden was determined in the urine, catheterized bladder, kidneys, and spleen. Data represent the log_10_ CFU/gram of tissue or the log_10_ CFU/ml of urine and are displayed as box-and-whisker plots with all data points shown; the horizontal middle lines indicate the mean. *, *P < *0.05; **, *P < *0.01; ***, *P < *0.001 (compared to the wild type by the nonparametric Mann-Whitney test). The numbers provided below each data set represent the percentage of mice that exhibited colonization above the limit of detection (100 CFU/gram of tissue or/ml of urine). ^, *P < *0.05; ^̂, *P < *0.01 (by chi-square test).

The experimental design of Tn-Seq is aimed at uncovering genes that contribute to the relative fitness of a bacterium competed against thousands of other mutants. However, fitness factors that are ideal targets for potential therapeutic intervention would also be expected to exhibit a defect during an independent challenge. We therefore utilized an independent challenge setup to assess colonization of each mutant that exhibited a significant fitness defect during single-species infection (*fliC*, *yscI*, *clpP*, *ddlA*, and *tatC*) for comparison to wild-type BE2467 (Fig. 14).

While none of the mutants were completely cleared from the urinary tract, four of five showed significant colonization defects in at least one organ: the *yscI* mutant exhibited reduced kidney and spleen colonization, the *clpP* mutant exhibited reduced bladder colonization, the *ddlA* mutant exhibited reduced bladder and spleen colonization, and the *tatC* mutant exhibited a defect in all organs. Infection with the *tatC* mutant also resulted in colonization levels at or below the limit of detection in a significant proportion of mice, while infection with the other three mutants with colonization defects resulted only in a significant decrease in the proportion of mice with bacteremia. Based on these observations, disruption of *fliC* appears to have impacted relative fitness only during cochallenge against the parental isolate, while loss of the other fitness factors also resulted in some degree of colonization defect independently of competition against the wild-type isolate. Factors such as *ddlA* and *tatC* may therefore represent preferable targets for potential therapeutic intervention.

## DISCUSSION

P. stuartii is a clinically relevant opportunistic pathogen with a high prevalence of antibiotic resistance, and yet little is known regarding the pathogenesis of this organism during infection. Here, we utilized Tn-Seq to identify P. stuartii genes that are essential for growth under standard laboratory conditions and to identify fitness factors for single-species versus polymicrobial CAUTI. Major strengths of this study included the following: (i) use of a genetically tractable CAUTI isolate with an available genome sequence (23), (ii) use of multiple transposon library pools and multiple mice per infection pool, (iii) stringent cutoffs for analysis of insertion site reads, and (iv) assessment of the impact of coinfection on fitness requirements. Limitations of the study included inability to assess the fitness contribution of genes that were poorly represented in the input pools, inability to accurately assess the fitness contribution of genes encoding secreted factors under conditions in which loss can be complemented by other mutants within the input pools, and potential polar effects of transposon insertions within operons, all of which are common limitations of Tn-Seq studies.

Despite these limitations, our approach successfully uncovered numerous P. stuartii fitness determinants for single-species and polymicrobial CAUTI, along with subsets of genes that contribute to fitness only during infection with each distinct type. By generating mutants in genes of interest and assessing fitness during direct cochallenge with wild-type P. stuartii, 6/7 (86%) fitness factors tentatively identified by Tn-Seq were validated as contributing to single-species CAUTI and 3/5 (60%) during coinfection with P. mirabilis. Furthermore, 4/5 fitness factors tested also exhibited colonization defects compared to wild-type P. stuartii in an independent challenge model, indicating that they may represent valid targets for therapeutic intervention.

There are several potential explanations for the failure of certain mutants to achieve validation during direct cochallenge, including the overall level of coverage of TA insertion sites within these genes, the specific location of the transposon insertions in a gene versus replacement of the majority of the gene with a hygromycin resistance cassette for generation of the mutants, the fold change and *P* value cutoffs used in the analysis, and potential polar effects of transposon insertions. It is also possible that some of the mutants might exhibit significant defects only when dramatically underrepresented in the input inoculum, such as in the case of the ∼1:10,000 ratio that would have been present during the Tn-Seq screen but not during a 1:1 cochallenge. Regardless of the source of discrepancy, the validation rate resulting from combined assessments of mutants during single-species and polymicrobial infection was 9/12 (75%), which is consistent with the validation rates reported in other Tn-Seq studies (ranging from 75% to 86%) (27, 29, 48–51).

An additional consideration regarding the validation experiments is that the mutagenesis strategy utilized to construct targeted mutants of interest has the potential to result in polar effects due to replacement of the gene with a hygromycin cassette. Of the seven genes chosen for validation studies, only three, *tatC*, *yscI*, and *impJ*, are present in operons; among the three, *tatC* is the final gene in the operon and unlikely to exhibit polar effects whereas *yscI* and *impJ* are in the middle of their respective operons. It is therefore possible that disruption of *yscI* and *impJ* would also result in loss of downstream gene products involved in their respective secretion systems, which might contribute to the fitness defects observed for the mutants. Future investigations of the role of each secretion system during P. stuartii CAUTI will therefore need to include an assessment of the relative contributions of these genes in production of a functional secretion apparatus.

In conclusion, P. stuartii carries a core set of genes and pathways that contribute to colonization during both single-species and polymicrobial infection, and those genes and pathways are both prevalent and highly conserved among isolates. Further research is needed to determine the *in vivo* importance of these core genes and pathways during both single-species and polymicrobial CAUTI, including coinfections with other common cocolonizing species. The underlying mechanisms of these core fitness requirements likely represent the most promising candidates for development of potential therapeutic interventions aimed at reducing P. stuartii colonization or minimizing risk of progression to severe infection and urosepsis. Furthermore, continued genome-wide assessment of multiple uropathogens is expected to uncover fitness factors shared between bacterial species, thereby elucidating common pathways for colonization and persistence.

## MATERIALS AND METHODS

### Ethics statement.

All animal protocols were approved by the Institutional Animal Care and Use Committee (IACUC) at the University of Michigan Medical School (PRO00005052) and the University of Buffalo (MIC31107Y), in accordance with the guidelines specified by the Office of Laboratory Animal Welfare (OLAW) and the United States Department of Agriculture (USDA), as well as guidelines specified by the Association for Assessment and Accreditation of Laboratory Animal Care, International (AAALAC, Intl.). Mice were anesthetized with a weight-appropriate dose (0.1 ml for a mouse weighing 20 g) of ketamine/xylazine (80 to 120 mg ketamine/kg of body weight and 5 to 10 mg xylazine/kg) by IP injection. Mice were euthanized by inhalant anesthetic overdose (Michigan) or CO_2_ asphyxiation (Buffalo), followed by vital organ removal.

Providencia stuartii isolates were collected from the urine of individuals with indwelling urinary catheters in a study approved by the University at Buffalo Institutional Review Board (STUDY00002526). All participants (or approved decision makers) provided written informed consent prior to initiation of investigation, and all participants also assented to being in the study.

### Bacterial strains and culture conditions.

Providencia stuartii strains BE2467, BE125, HI122, KU146, DE266, RO106, GO42, MC118, BR143, TA101, NI114, and 955 and Proteus mirabilis strain HI4320 are part of a strain collection of urine isolates that were previously obtained from the urine of catheterized nursing home residents (10, 23, 52). P. stuartii strains 101v0MAC2, 101v0MAC3, 201v0MAC1, 202v0MAC2, and 206v0MAC4 were isolated from the urine of catheterized nursing home residents under STUDY00002526, and the isolates were verified to represent Providencia stuartii by the use of API-20E test strips (bioMérieux, Marcy-l’Étoile, France).

Bacteria were routinely cultured at 37°C with aeration in 5 ml low-salt LB broth (10 g/liter tryptone, 5 g/liter yeast extract, 0.5 g/liter NaCl) or on low-salt LB medium solidified with 1.5% agar. Transposon mutants were cultured in LB medium containing 150 μg/ml hygromycin (Research Products International). Additional P. stuartii mutants were constructed for validation of conditionally essential genes by the use of a previously described allelic exchange vector to replace the gene of interest with a hygromycin resistance cassette (27). Resulting mutants were screened by hygromycin selection and PCR (all primers used in this study for generation and verification of mutants are listed in Table S2 in the supplemental material).

10.1128/mSphere.00412-20.6 TABLE S2 Primers used in this study. Download Table S2, XLSX file, 0.01 MB. Copyright © 2020 Johnson et al. 2020 Johnson et al. This content is distributed under the terms of the Creative Commons Attribution 4.0 International license.

### Construction of pAOJ12.

To generate a transposon vector suitable for mutagenesis in P. stuartii, the hygromycin resistance cassette from pSIM18 (53) was subjected to PCR amplification with primers AOJ_123 and AOJ_124 and cloned into pCR2.1-TOPO (Thermo Fisher Scientific) to create pAOJ8. Plasmid pAOJ8 was digested with MfeI and SpeI restriction enzymes and cloned between the XbaI and MfeI sites on plasmid pSAM_Ec (54), resulting in the hygromycin resistance cassette being flanked by Mariner transposon inverted repeats. The resulting plasmid was named pSAM_Hygromycin.

A conditionally replicating plasmid was made by digesting pRK415 (55) with MfeI and religating, removing an ∼2.8-kb fragment containing the *trfA1*/*A2* replication protein open reading frames. This plasmid, pRK415ΔMfeI, and all derivatives were transformed into E. coli EPI300, which expresses the TrfA protein under the control of the arabinose promoter (56), and were cultured in media containing 0.5% arabinose for propagation. Next, three fragments were amplified as follows: (i) the temperature-sensitive λcI857 repressor from pSIM18, using primers AOJ_173/AOJ_174; (ii) the Himar 1C9 transposase from pSAM_Ec, using AOJ_175/AOJ_176; and (iii) the strong rho-independent *spy* transcriptional terminator from E. coli Top 10 (57), using AOJ_177/AOJ_178. These fragments were stitched together into a single unit using NEBuilder Hifi DNA assembly master mix. The entire assembled fragment was amplified using AOJ_173 and AOJ_178, which also contain NheI and MfeI restriction sites. The AOJ_173/AOJ_178 amplified fragment was digested with these two enzymes and ligated into the EcoRI-XbaI site of pRK415ΔMfeI to generate pAOJ11. To insert the λP_L_ promoter into pAOJ11, an ∼300-bp fragment was created by PCR amplification of pSIM18 with primers AOJ_195/AOJ_196, which add NdeI and KpnI sites, allowing this fragment to be ligated into the NdeI-KpnI site of pAOJ11 to make pAOJ11+P_L_. Finally, pAOJ11+P_L_ and pSAM_Hygromycin were digested with BamHI and KpnI, and the pSAM_Hygromycin fragment containing the transposon was ligated into pAOJ11+P_L_ to make pAOJ12.

Once verified by restriction digestion, pAOJ12 was transferred into E. coli S17-1λpir for biparental mating. Plasmid pAOJ12 contains an RK2-based suicide origin that can replicate only with TrfA supplied in *trans*, an RK2 origin of transfer for mating, a hygromycin resistance transposon, and a hyperactive Himar1C9 transposase to drive transposition when expression is induced from the λP_L_ promoter by temperature elevation. As the λP_L_ promoter is active in many distally related Gram-negative bacteria, the RK2 conjugation system can transport DNA into a large variety of species, and hygromycin B has the useful features of being broadly toxic but not used clinically. Thus, we expect that this transposon vector could be useful for transposon mutagenesis in many Gram-negative species beyond P. stuartii.

### Generation and validation of transposon mutants.

P. stuartii BE2467 and E. coli S17-1λpir(pAOJ12) were cultured overnight in LB medium containing appropriate antibiotics at 30°C with aeration at 225 rpm. P. stuartii BE2467 (250 μl) and E. coli S17-1λpir(pAOJ12) (500 μl) were inoculated into separate conical tubes, each containing 25 ml fresh LB medium prewarmed to 30°C, with antibiotics appropriate for S17-1λpir(pAOJ12). Cultures were incubated with shaking for 2 h at 30°C before centrifugation to pellet was performed, and the cell pellets were washed once with phosphate-buffered saline (PBS; 0.128 M NaCl, 0.0027 M KCl, pH 7.4). The cell pellets were resuspended in 0.5 ml PBS, combined into a single microcentrifuge tube, and centrifuged to pellet. The supernatant was removed, and the pellet consisting of the combined cells was incubated at ambient temperature for 5 min, after which the pellet was gently resuspended in 250 μl LB medium, spread onto a sterile 0.45-μm-pore-size filter resting on a LB agar plate, and incubated at 30°C for 90 min. Next, the mating mixture was removed from the filter and placed into 2.5 ml LB medium with 10 μg/ml chloramphenicol (stopping the growth of E. coli) and heated to 42°C for a 10-min heat shock. The culture was then transferred to 37°C with shaking to recover for 90 min, followed by plating on LB medium containing 150 μg/ml hygromycin B and 10 μg/ml chloramphenicol. Inoculated plates were incubated overnight at 37°C, and colonies were enumerated the following day. Roughly 50,000 colonies were scraped from the resulting plates into 5 pools of 10,000 colonies each for generation of glycerol freezer stocks, which were frozen at –80°C until further use.

The randomness of the insertions was verified by extracting genomic DNA from subcultures of individual colonies, digesting with EcoRV, and Southern blotting with a digoxigenin (Roche)-labeled probe targeting the hygromycin resistance cassette within the transposon, which was amplified by primers AOJ_199 and AOJ 200 (see Fig. S1 in the supplemental material).

### Mouse model of CAUTI.

Infection studies were carried out as previously described (21, 27) using a modification of the Hagberg protocol (58). Transposon mutant pools (1 ml volume) were thawed in 9 ml fresh LB medium with hygromycin and cultured at 37°C for no more than 10 h. Cultures were adjusted to an optical density at 600 nm (OD_600_) of ∼0.2 (2 × 10^8^ CFU/ml) and were diluted 1:100. Female CBA/J mice (Envigo or Jackson Laboratory) (6 to 8 weeks of age) were inoculated transurethrally with 50 μl of 2 × 10^6^ CFU/ml (1 × 10^5^ CFU/mouse), and a 4-mm-long segment of sterile silicone tubing (Braintree Scientific, Inc.) (0.64-mm outer diameter [O.D.], 0.30-mm inner diameter [I.D.]) was carefully advanced into the bladder during inoculation and retained for the duration of the study as described previously (23, 59–61).

For coinfection experiments, mice were inoculated with 50 μl of a 1:1 mixture of the P. stuartii transposon mutant pools and WT P. mirabilis HI4320. For each transposon pool input, 5 to 10 mice were inoculated for single-species infection and 5 to 10 mice were coinfected with P. mirabilis. Mice were euthanized at 4 days postinoculation (dpi), and bladders, kidneys, and spleens were harvested into PBS. Notably, catheter segments were not removed from the bladder samples for homogenization, so CFU data for bladder samples represent a catheterized bladder. Tissues were homogenized using either an Omni TH homogenizer (Omni International) or a Bullet Blender 5 Gold homogenizer (Next Advance), and a 150-μl aliquot was removed and plated using either an Autoplate 4000 spiral plater (Spiral Biotech) or an EddyJet 2 spiral plater (Neutec Group) for enumeration of colonies using either a QCount automated plate counter (Spiral Biotech) or a ProtoCOL3 automated colony counter (Synbiosis). The remaining bladder and kidney homogenates were spread plated in their entirety, and colonies were collected, pelleted, and frozen for sequencing. A competitive index (CI) value was calculated for all samples in which bacterial burden was described as being above the limit of detection as follows:

$$\text{CI }=\frac{\text{Strain A output}/\text{Strain B output}}{\text{Strain A input}/\text{Strain B input}}$$

A log_10_CI value of 0 indicates that the ratio of the strains in the output was similar to that in the input and that neither strain had an advantage. A log_10_CI value of >0 indicates that strain A had a competitive advantage over strain B. A log_10_CI value of <0 indicates that strain B had a competitive advantage over strain A.

For independent challenge experiments, groups of 5 to 6 mice were inoculated with 50 μl of 2 × 10^6^ CFU/ml (1 × 10^5^ CFU/mouse) of a strain of interest, and a catheter segment was inserted as described above. Mice were euthanized 4 days postinoculation (dpi) as described above, and organ homogenates were plated for enumeration of colonies.

### Illumina sequencing.

Genomic DNA was isolated from the five input inocula, with two technical replicates each, and from the bladder and kidney homogenates of each individual mouse (outputs) by cetyltrimethylammonium bromide (CTAB) precipitation (62). Samples were enriched for transposon insertion junctions as outlined previously by Goodman et al. (63), and concentration and purity were confirmed by TapeStation analysis. Samples were multiplexed and subjected to V4 Single-End 50 HiSeq-2500 high-output sequencing as follows: (i) 5 input samples with 2 replicates each were multiplexed and sequenced on a single lane, (ii) P. stuartii single-species outputs from 20 mouse bladders were multiplexed and sequenced on two lanes, (iii) P. stuartii single-species outputs from 20 mouse kidneys were multiplexed and sequenced on two lanes, (iv) outputs from P. stuartii coinfection with P. mirabilis from 20 mouse bladders were multiplexed and sequenced on two lanes, and (v) outputs from P. stuartii coinfection with P. mirabilis from 20 mouse kidneys were multiplexed and sequenced on two lanes. Each lane was spiked with 15% bacteriophage φX DNA to overcome effects of the presence of low-diversity sequences. Sequencing was performed at the University of Michigan DNA core facility. All raw sequence reads are available in the Sequence Read Archive under BioProject PRJNA578390 as accession numbers SAMN13059443 to SAMN13059447 and SRA accession numbers SRR10312361 to SRR10312365. The barcodes associated with each unique sample are provided in Table S3.

10.1128/mSphere.00412-20.7 TABLE S3 Barcodes for demultiplexing raw Tn-Seq data. Download Table S3, XLSX file, 0.01 MB. Copyright © 2020 Johnson et al. 2020 Johnson et al. This content is distributed under the terms of the Creative Commons Attribution 4.0 International license.

### Mapping of transposon insertion sites.

The chromosome and plasmid sequences of P. stuartii BE2467 (NCBI accession numbers CP017054, CP017055, and CP017056) (23) were combined into a single genome sequence with 1,000 N’s added between each pair of contigs, and the coordinates of genes were adjusted accordingly. The Goodman In-Seq pipeline (63) was applied on the raw reads to perform read filtration, transposon nucleotide removal, debarcoding, alignment, and insertion calling, following a script that was previously used to map insertions onto the P. mirabilis genome (27, 29).

### Identification of essential genes.

Essential genes were identified as described previously (27). Briefly, input samples were corrected for potential read count bias, and a Bayesian mixture model was used to estimate the rate for insertion counts in each gene by assuming that the counts followed a Poisson distribution. JAGS (64) was used to obtain a posterior probability estimate for evaluating the likelihood of each gene being essential. Genes identified as having a 90% probability or greater of being essential were classified as “essential genes.” Protein sequences of estimated essential genes were annotated for orthologous groups (GO terms, KO terms, and COG categories) using the eggNOG-mapper function of EggNOG version 4.5.1 via HMMer search restricted to the “bacteria” HMM database (65).

### Identification of candidate fitness factors for single-species and polymicrobial CAUTI.

Fitness factors were identified using the approach previously published for P. mirabilis (27, 29). Briefly, the output samples from 20 mice (including 4 mice per input pool) were combined for estimation of the fitness contribution to a given organ for a given infection type (bladder versus kidneys and single-species versus coinfection). Individual genes were assessed for fitness contribution only when the mean of the sum of insertion site reads was >1,000 and the number of insertions in that gene was >5. The fitness contribution of each gene was then estimated as previously described (27, 29) using an R package called Tn-SeqDiff (66), which can be installed from the Comprehensive R Archive Network (CRAN). Significant genes for further analysis were selected based on an adjusted *P* value of <0.05 and a >20-fold ratio of input over output.

### Proteome analysis.

To identify homologs of candidate core fitness factors first, a file was generated containing the FASTA sequences of each fitness factor and then the proteomes of other P. stuartii strains available from the Pathosystems Resource Integration Center (PATRIC) were compared to that sequence using the PATRIC proteome comparison tool (67). The sequence identity was limited to ≥10% over a minimum of 30% sequence for this comparison.

### Growth curves.

Overnight cultures of P. stuartii mutants washed 1× in PBS and diluted 1:100 in growth medium, and 100-μl aliquots were dispensed into at least 4 replicate wells of a 96-well plate. A BioTek Synergy H1 96-well plate reader was utilized to generate growth curves during incubation at 37°C with continuous double-orbital shaking for aeration. OD_600_ readings were taken every 15 min for 18 h.

### RNA isolation.

P. stuartii BE2467 was cultured overnight at 37°C with shaking at 225 rpm in LB broth with 25 μg/ml kanamycin. A 500-μl volume of the culture was centrifuged to pellet, resuspended in 0.5 ml of sterile saline solution (0.9% NaCl [wt/vol]), and washed twice. The final suspension was diluted 1:100 into 5 ml of prewarmed filter-sterilized human urine or fresh LB medium and incubated for 2 h at 37°C with shaking at 225 rpm. Four 1-ml aliquots were then removed, centrifuged to pellet, and resuspended in 1 ml of RNA STAT-60 (Tel-Test, Inc.). Chloroform-isoamyl alcohol (Calbiochem) (24:1; 0.2 ml) was added to each aliquot, and microcentrifuge tubes were inverted several times for mixing and then centrifuged at 18,000 × *g* for 30 min at 4°C. The aqueous layer was removed and transferred to a new microcentrifuge tube, to which 0.5 ml isopropanol was added (Fisher Scientific), and the reaction mixture was then mixed by inversion and incubated overnight at –20°C to precipitate RNA. The next day, samples were centrifuged at 18,000 *× g* for 30 min at 4°C, supernatants were discarded, and the nucleic acid pellet was washed three times with 1 ml 75% ethanol and centrifuged at 18,000 × *g* for 10 min after each wash. After the final wash, the supernatant was carefully removed and the pellet was air-dried and then dissolved in 50 μl of molecular-biology-grade water (Corning). The nucleic acid concentration was measured by the use of a NanoDrop instrument and adjusted with water to 200 ng/μl in a 50-μl total volume. DNA was removed with two separate rounds of digestion via the use of an Invitrogen DNA-free kit, according to the manufacturer’s protocol. DNase-treated RNA was frozen at −20°C until use.

### cDNA synthesis and RT-qPCR.

A 1-μg volume of DNase-treated RNA in a total volume of 20 μl was used to generate cDNA via the use of an iScript cDNA synthesis kit (Bio-Rad) per manufacturer’s instructions. After cDNA synthesis, sample volumes were increased to 40 μl by adding 20 μl TE buffer (10 mM Tris [pH 8], 1 mM EDTA), and cDNA was frozen at –20°C until use. Three technical replicate reaction mixtures for each condition were prepared by combining 1 μl cDNA (approximately 25 ng of RNA template), 2 μl primers (10 pmol/μl), 7 μl water, and 10 μl qPCRBio SyGreen Blue Mix Lo-Rox (PCR Biosystems). Reactions were initiated on a Bio-Rad CFX-Connect real-time system with the default CFX reverse transcriptase quantitative PCR (RT-qPCR) protocol. For analysis, *recA* was chosen as the reference gene for normalization between samples as it exhibited a low level of variation between the urine and LB samples. Data were analyzed according to the relative quantification (RQ) method as described previously by Pfaffl (68), in which LB medium was considered the “Control” growth condition and urine was considered the “Sample” growth condition, in the following equation:

$$\text{Relative Quantification ratio }=\frac{{\left(E_{\text{target}}\right)}^{\Delta \text{CPtarget }(\text{control}-\text{sample})}}{{\left(E_{\text{reference}}\right)}^{\Delta \text{CPrefernce }(\text{control}-\text{sample})}}$$

In this equation, *E* refers to the primer efficiency of the target gene (*E*_target_) or the reference gene (*E*_reference_), and CP refers to the cycle point, or the cycle number at which the signal exceeds the threshold.

### Motility assay.

Swimming motility agar (MOT) plates (10 g/liter tryptone, 5 g/liter yeast extract, 5 g/liter NaCl, 3 g/liter agar) were subjected to stab inoculation with an overnight culture of P. stuartii BE2467 or the isogenic *fliC* mutant. MOT plates were incubated without inversion at 37°C for 7 days for assessment of flagellin-mediated motility. In addition, overnight cultures of P. stuartii strains of interest were subjected to stab inoculation into motility test medium with triphenyltetrazolium chloride (TTC; Hardy Diagnostics) and incubated 37°C for 24 h. In this medium, motile bacteria extend outward from the stab line and reduce the TTC, generating a diffuse red pigment, while nonmotile organisms grow only along the stab line, generating a concentrated line of red pigment and leaving the surrounding medium clear.

### *fliA* sequence investigation.

Sequences of the *fliA* gene of P. stuartii BE2467 and six other P. stuartii isolates were obtained from the PATRIC database and aligned using the free sequence alignment tool from Benchling (benchling.com). Genomic DNA was extracted from P. stuartii BE2467 and two motile revertants, and *fliA* was subjected to PCR amplification using primers AOJ_325 and AOJ_326 (Table S2) and to Sanger sequencing for alignment with the other *fliA* sequences in Benchling. The resulting alignment is displayed in Fig. S4.

### Assessment of pigment production.

Overnight cultures of wild-type P. stuartii BE2457 and the isogenic *tatC* mutant were subjected to drip plating onto non-low-salt LB agar (10 g/liter tryptone, 5 g/liter yeast extract, 10 g/liter NaCl, 1.5% agar) supplemented with 0.4 g/liter tyrosine and 0.25 g/liter iron (II) sulfate heptahydrate. Pigment production was visually assessed after incubation for ∼18 h at 37°C.

### Assessment of growth on MacConkey agar.

Overnight cultures of P. stuartii BE2467 and the isogenic *tatC* mutant were adjusted to an OD of 2.0 (2 × 10^9^ CFU/ml), subjected to serial dilution, and plated onto MacConkey agar. Viable bacteria were enumerated after incubation for ∼18 h at 37°C or 30°C.

### Assessment of heat sensitivity.

P. stuartii BE2467 and the isogenic *clpP* mutant were cultured overnight in LB broth at 37°C with aeration. Cultures were then adjusted to an OD of 2.0 (2 × 10^9^ CFU/ml) and subjected to serial dilution, and 10 μl of each dilution was spotted onto LB agar. Plates were incubated at either 37°C or 45°C for 20 h prior to growth assessment.

### MIC of d-cycloserine.

P. stuartii BE2467 and the isogenic *ddlA* mutant were cultured overnight in LB broth at 37°C with aeration. Cultures were adjusted to an OD of 0.2 (2 × 10^8^ CFU/ml) and incubated with increasing concentrations of d-cycloserine for 18 h at 37°C with aeration, followed by measurement of optical density at 600 nm. For cultures with an OD_600_ reading of >1.0, the culture was diluted 1:10 and remeasured, and the reading was then multiplied by the dilution factor.

### Macrophage-like cell survival assay.

Survival rates of wild-type P. stuartii BE2467 and the *yscI* mutant were measured using the RAW 264.7 murine macrophage-like cell line for an antibiotic protection assay, as described previously (51). RAW cells were cultured in antibiotic-free RPMI 1640–10% heat-inactivated fetal bovine serum. Amikacin was used at a concentration of 100 μg/ml in place of gentamicin due to the high level of gentamicin resistance observed for P. stuartii BE2467. Following antibiotic treatment, the RAW cells were lysed with 1% saponin–PBS (pH 7.4) to quantify viable intracellular bacteria.

### Statistical analysis.

Significance was assessed using a combination of two-way analysis of variance (ANOVA), Student's *t* test, the nonparametric Mann-Whitney test, and the Wilcoxon signed-rank test. These analyses were performed using GraphPad Prism, version 7 (GraphPad Software, San Diego, CA). All *P* values are two-tailed at a 95% confidence interval.

### Data availability.

Tn-Seq raw reads are freely available in the Sequence Read Archive under BioProject PRJNA578390 as accession numbers SAMN13059443 to SAMN13059447 and SRA accession numbers SRR10312361 to SRR10312365, and the complete data set is listed in Table S1.
