# Supplementary figures and images for: Transposon Insertion Site Sequencing of Providencia stuartii: Essential Genes, Fitness Factors for Catheter-Associated Urinary Tract Infection, and the Impact of Polymicrobial Infection on Fitness Requirements
Source: mSphere. 2020 May 27;5(3):e00412-20. doi: 10.1128/mSphere.00412-20 (PMC7253602; doi:10.1128/mSphere.00412-20)

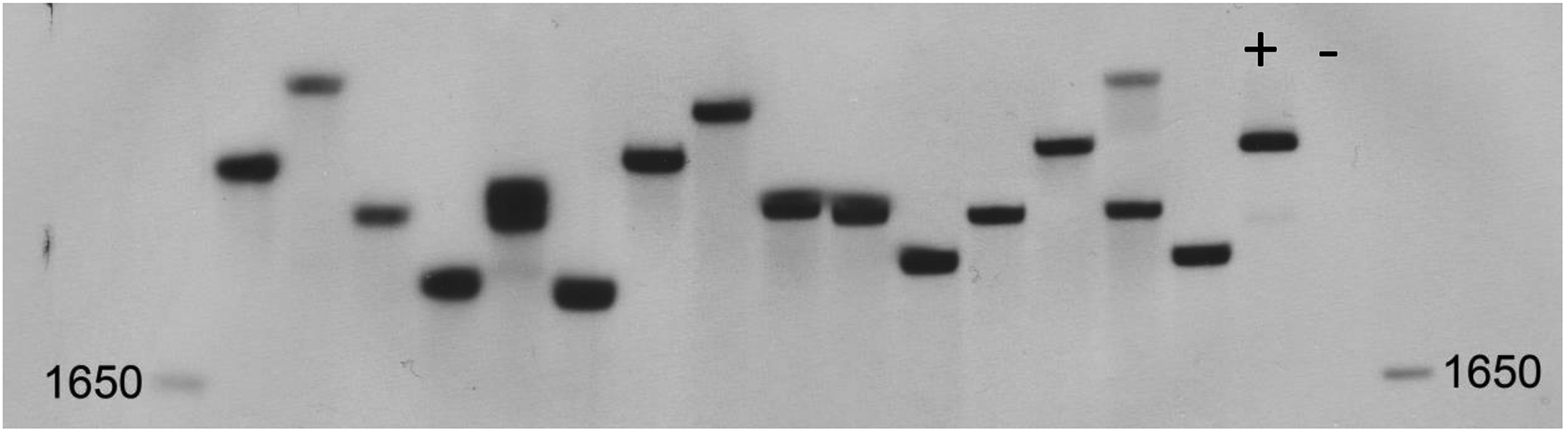

Supplement: FIG S1 [file mSphere.00412-20-sf001.tif]

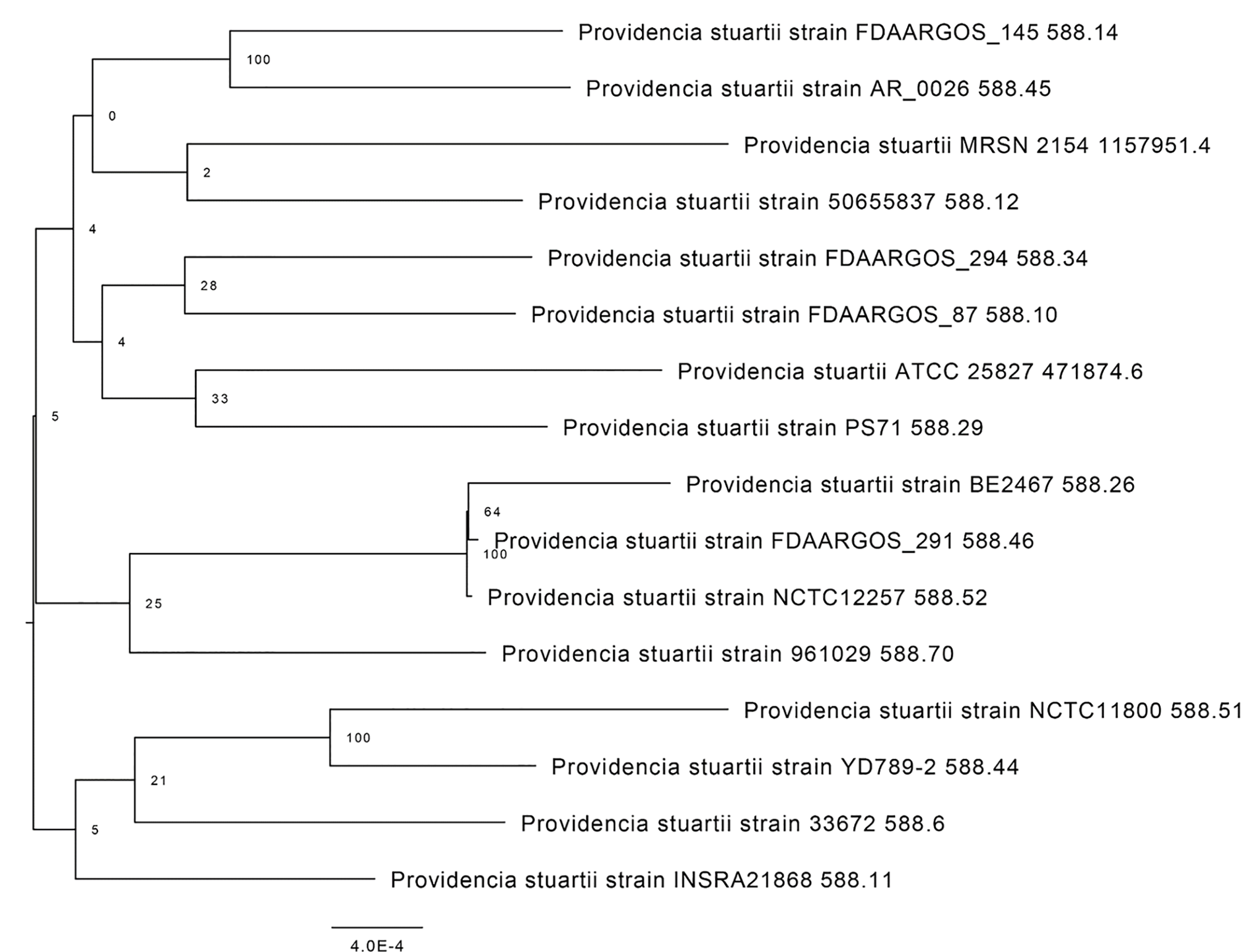

Supplement: FIG S2 [file mSphere.00412-20-sf002.tif]

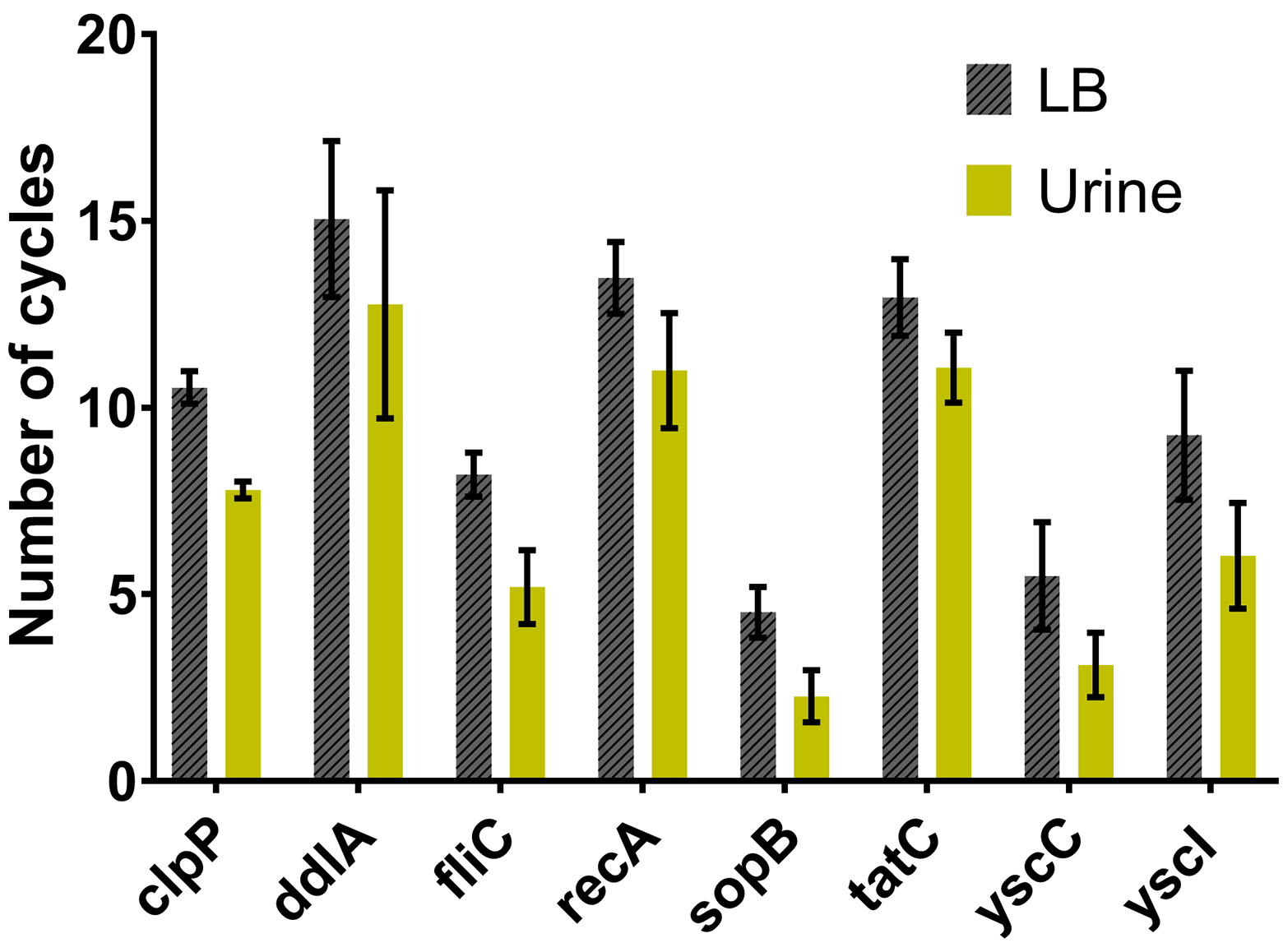

Supplement: FIG S3 [file mSphere.00412-20-sf003.tif]

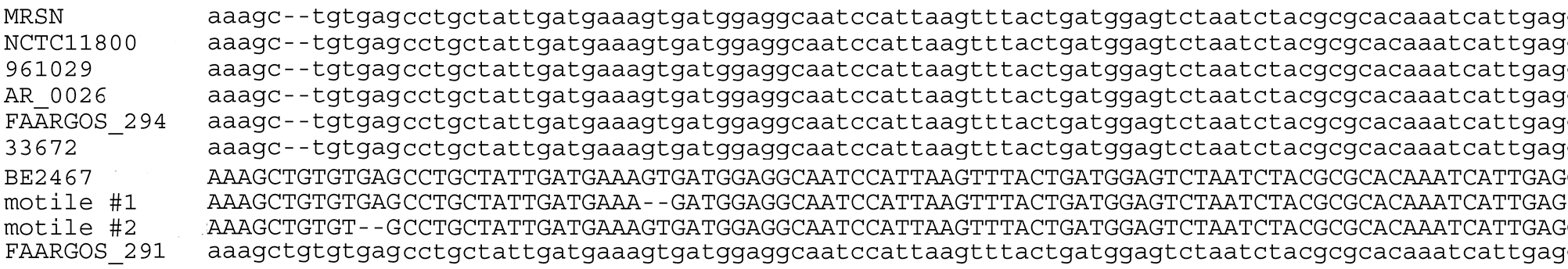

Supplement: FIG S4 [file mSphere.00412-20-sf004.tif]
